# Neutrophil Extracellular Traps Regulate Surgical Brain Injury by Activating the cGAS-STING Pathway

**DOI:** 10.1007/s10571-024-01470-9

**Published:** 2024-04-18

**Authors:** Bingbing Li, Lixia Xu, Zhengang Wang, Qi Shi, Yang Cui, Weijia Fan, Qiaoli Wu, Xiaoguang Tong, Hua Yan

**Affiliations:** 1 Clinical College of Neurology, Neurosurgery and Neurorehabilitation, Tianjin Medical University, Tianjin, 300070 China; 2 Tianjin Key Laboratory of Cerebral Vascular and Neurodegenerative Diseases, Tianjin Neurosurgical Institute, Tianjin Huanhu Hospital, Tianjin, 300350 China; 3 Department of Neurosurgery, Tianjin Huanhu Hospital, Tianjin, 300350 China

**Keywords:** Surgical brain injury, Neutrophil extracellular traps, cGAS, STING, Vitamin C

## Abstract

Surgical brain injury (SBI), induced by neurosurgical procedures or instruments, has not attracted adequate attention. The pathophysiological process of SBI remains sparse compared to that of other central nervous system diseases thus far. Therefore, novel and effective therapies for SBI are urgently needed. In this study, we found that neutrophil extracellular traps (NETs) were present in the circulation and brain tissues of rats after SBI, which promoted neuroinflammation, cerebral edema, neuronal cell death, and aggravated neurological dysfunction. Inhibition of NETs formation by peptidylarginine deiminase (PAD) inhibitor or disruption of NETs with deoxyribonuclease I (DNase I) attenuated SBI-induced damages and improved the recovery of neurological function. We show that SBI triggered the activation of cyclic guanosine monophosphate–adenosine monophosphate synthase stimulator of interferon genes (cGAS-STING), and that inhibition of the cGAS-STING pathway could be beneficial. It is worth noting that DNase I markedly suppressed the activation of cGAS-STING, which was reversed by the cGAS product cyclic guanosine monophosphate–adenosine monophosphate (cGMP-AMP, cGAMP). Furthermore, the neuroprotective effect of DNase I in SBI was also abolished by cGAMP. NETs may participate in the pathophysiological regulation of SBI by acting through the cGAS-STING pathway. We also found that high-dose vitamin C administration could effectively inhibit the formation of NETs post-SBI. Thus, targeting NETs may provide a novel therapeutic strategy for SBI treatment, and high-dose vitamin C intervention may be a promising translational therapy with an excellent safety profile and low cost.

**Graphical Abstract:**

The schematic diagram shows the formation of NETs activated cGAS-STING pathway after SBI, leading to increased microglia activation, accompanied with elevation of inflammatory factors, which in turn aggravated brain injury.

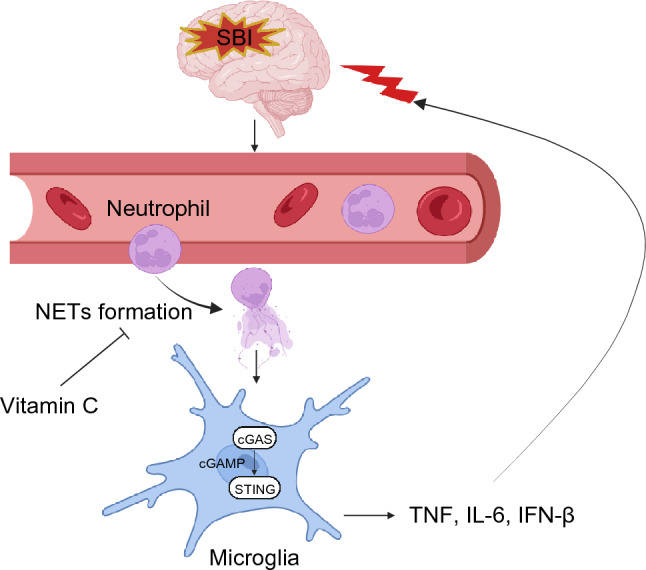

**Supplementary Information:**

The online version contains supplementary material available at 10.1007/s10571-024-01470-9.

## Introduction

Approximately 13.8 million patients worldwide require surgery each year due to traumatic brain injury, stroke-related conditions, tumors, hydrocephalus, and epilepsy (Dewan et al. 2018). Surgical brain injury (SBI), also known as surgically-induced brain injury, is unavoidable brain damage caused by neurosurgical procedures or surgical instruments during neurosurgery (Jadhav et al. 2007; Kim et al. 2017; Wang et al. 2018). Although modern science has reduced the damage caused by invasiveness, SBI can still lead to postoperative complications such as neuroinflammation, cerebral edema, neuronal cell death, and aggravate neurological impairment, all of which tend to be detrimental to the postoperative rehabilitation of patients (Wang et al. 2018; Zagzoog and Reddy 2020). However, SBI is often underappreciated by neurosurgeons and neurologists. Currently, the pathophysiological mechanisms of SBI have not yet been fully clarified and there is still a lack of effective therapies directly targeting SBI.

Neutrophils, an important component of the innate immune system, play a two-sided role: friend and foe (Liu et al. 2018; Sas et al. 2020). Studies have shown that neutrophil infiltration into brain tissues can promote neuroinflammation and cerebral edema and that depletion of neutrophils can alleviate these adverse effects (Liu et al. 2018; Strecker et al. 2017; Harris et al. 2005). However, depletion or inhibition of neutrophils may carry a greater risk of infection, especially in postsurgical patients or critically ill patients (Lewis et al. 2013; Vaibhav et al. 2020).

Neutrophil extracellular traps (NETs) are produced by activated neutrophils and are composed of double-stranded DNA, histones, and other proteins (Papayannopoulos 2018). They have attracted a great deal of attention (Papayannopoulos 2018; Zhao et al. 2023). In recent years, NETs have been shown to play an important role in the pathologies of acute central nervous system (CNS) diseases, such as traumatic brain injury (Vaibhav et al. 2020; Liu et al. 2022), subarachnoid hemorrhage (Hanhai et al. 2021; Zeng et al. 2022), and ischemic stroke (Kim et al. 2019; Wang et al. 2021; Denorme et al. 2022).

Cyclic GMP-AMP synthase (cGAS) is a crucial cytosolic DNA sensor that recognizes double-stranded DNA (dsDNA) (Wu et al. 2013). The binding of cGAS to cell-free DNA generates the second messenger 2′-3′-cGAMP, which subsequently binds and activates the stimulator of interferon genes (STING), thereby inducing the production of type I interferon (IFN) (Chen et al. 2016). The cGAS-STING pathway is associated with cytoplasmic dsDNA-triggered inflammatory responses (Gaidt et al. 2017). Studies suggest that NETs are potent inducers of cGAS, and NETs may be involved in the regulation of inflammatory injury via the cGAS-STING signaling pathway (Apel et al. 2021; Wang et al. 2021).

Although the pathophysiological roles of NETs and cGAS-STING have been studied for years, no studies of their roles in SBI are available to date. An increasing number of studies suggest that targeting NETs or the cGAS-STING pathway may be a promising therapeutic strategy for acute CNS diseases (Denorme et al. 2022; Hu et al. 2022; Shao et al. 2023). However, its clinical translation will be challenging (Denorme et al. 2022).

Vitamin C is an essential vitamin that must be obtained through the diet in humans, and it has strong antioxidant activity (Harrison and May 2009). Vitamin C can be rapidly depleted in states of critical illness, and exogenous vitamin C intervention may be beneficial in patients with acute brain injury, but the exact mechanisms are not fully understood (Leichtle et al. 2020; Kangisser et al. 2021). Vitamin C has been shown to be a novel regulator of NETs formation, and this complements the notion that vitamin C is protective in sepsis (Mohammed et al. 2013). It is unclear whether vitamin C can inhibit the formation of NETs after SBI.

We hypothesized that the excessive production of NETs could play a detrimental role in the pathogenesis of SBI via the cGAS-STING pathway. Here, we explored whether the generation of NETs is induced by SBI in a rat model, and determined whether targeting NETs with Cl-amidine (a pan-PAD inhibitor) or DNase I could be neuroprotective after SBI. We uncovered that NETs promote neuroinflammation and cerebral edema, and aggravate neurological impairment by activating the cGAS-STING pathway. We also demonstrate that high-dose intravenous vitamin C inhibits the formation of NETs after SBI, providing a new idea for the treatment of SBI.

## Materials and Methods

### Animals

All experimental protocols were evaluated and approved by the institutional animal care committee of Tianjin Key Laboratory of Cerebral Vascular and Neurodegenerative Diseases (HHLL-2023-025) and complied with the ARRIVE guidelines. Male Sprague–Dawley (SD) rats (grade SPF; 8–9 weeks old, 270–320 g) were housed in the animal care facility (four rats per cage) on a constant 12-hour light/dark cycle with controlled temperature at Tianjin Huanhu Hospital (Tianjin, China). All SD rats had free access to food and water.

### Experimental Design

#### Experiment 1

Rats were randomly divided into six groups consisting of a sham group and five SBI subgroups (at 6 h, 12 h, 1 d, 3 d, and 7 d post-SBI). Plasma and samples of brain tissues were collected for the enzyme-linked immunosorbent assay (ELISA) (*n* = 6/group) and western blot (*n* = 6/group) detection respectively. Brain edema was evaluated using brain water content in six groups (*n* = 6/group). The surrounding brain tissues of injured foci and neutrophils isolated from the peripheral blood were observed by scanning electron microscopy (*n* = 5/group). Furthermore, brain tissues were obtained for immunofluorescence at 3 days post-modeling (*n* = 6/group).

#### Experiment 2

The rats were randomly divided into four groups: sham, SBI + vehicle (saline), SBI + Cl-amidine, and SBI + DNase I. Plasma and brain tissues of rats were obtained at 1 d and 3 d post-SBI respectively. ELISA (*n* = 6/group), western blot (*n* = 6/group), immunofluorescence (*n* = 6/group), brain water contents (*n* = 6/group), and terminal deoxynucleotidyl transferase dUTP nick-end labeling (TUNEL) (*n* = 6/group) were performed. The modified Garcia scores were used to assess neurological deficits in different groups at 1 day, 3 days, and 7 days (*n* = 8/group).

#### Experiment 3

Rats were randomly divided into two groups: SBI + vehicle (1% DMSO diluted in corn oil) group, and SBI + RU.521 group. The plasma samples were collected for ELISA (*n* = 6/group) at 1 day post-SBI. Brain tissues were obtained at 3 days post-SBI for ELISA (*n* = 6/group), brain water content (*n* = 6/group), and TUNEL analyses (*n* = 6/group) respectively. Neurological function was assessed by the modified Garcia scores at 1 day, 3 days, and 7 days after SBI (*n* = 8/group).

#### Experiment 4

Rats were randomly divided into four groups: sham, SBI + vehicle (saline), SBI + DNase I, and SBI + DNase I + cGAMP. Brain tissues were collected for western blot 3 days post-SBI (*n* = 6/group). Furthermore, rats were randomly divided into two groups: SBI + DNase I + vehicle (saline), SBI + DNase I + cGAMP. Brain tissues were collected 3 days post-SBI for immunofluorescence, TUNEL, and brain water content analyses respectively (*n* = 6/group). Neurological function was evaluated at 1 day, 3 days, and 7 days post-SBI (*n* = 8/group).

#### Experiment 5

Rats were randomly divided into three groups including a sham group, an SBI + vehicle (saline) group, and an SBI + DNase I group, and two groups containing SBI + DNase I + vehicle (saline) group, SBI + DNase I + cGAMP group. Neutrophils isolated from peripheral blood were obtained at 1 day after SBI. Primary rat microglia were cocultured with neutrophils isolated from different groups. Immunofluorescence (*n* = 6/group) and ELISA (*n* = 6/group) were performed.

#### Experiment 6

Rats were randomly divided into four groups: SBI + vehicle (saline) group, SBI + vitamin C (100 mg/kg) group, SBI + vitamin C (200 mg/kg) group, and SBI + vitamin C (500 mg/kg) group. The ROS levels of neutrophils isolated from peripheral blood were measured in different intervention groups (*n* = 6/group). Plasma and samples of brain tissues were collected for the ELISA (*n* = 6/group) and western blot detection (*n* = 6/group). Neutrophils isolated from normal healthy rats were pretreated with different concentrations of vitamin C (100 nM, 500 nM, 1µM) and then stimulated with phorbol 12-myristate 13-acetate (PMA). Live-cell-forming NETs were visualized by extracellular DNA (SYTOX Green) and intracellular DNA (Hoechst 33342) on laser confocal microscopy (*n* = 5/group).

### SBI Model

The SBI rat model was performed as previously described (Jadhav et al. 2007). Rats were anesthetized by intramuscular injection of a mixture of tiletamine/zolazepam (Zoletil 50, Virbac, France, 20 mg/kg) and xylazine hydrochloride (#T1500, TargetMol, China, 5 mg/kg). The skin and subcutaneous tissue were incised, and the right frontal skull was exposed. A bone flap was removed, the dura was incised, and a partial right frontal lobectomy was performed by making 2 incisions: 2 mm lateral to the sagittal suture and 1 mm proximal to the coronal suture, with depth extending to the skull base. Sham-operated animals were only subjected to craniotomy without any dural incisions. Then they were closely observed and transferred to cages. The rats were sacrificed at the indicated time points after surgery.

### Drug Administration

For drug administration, Cl-amidine (#T10831L, TargetMol, China) and DNase I (#11284932001, Roche Diagnostics, Germany) were diluted in sterile saline. As previously reported (Vaibhav et al. 2020; Feng et al. 2021), Cl-amidine (50 mg/kg) was administered via intraperitoneal injection 10 min after SBI, and DNase I (5 mg/kg) was administered via the tail vein at 1 h post-SBI. SBI rats of the vehicle groups were treated with a corresponding dose of saline. The administrations of Cl-amidine, DNase I, and saline were performed daily respectively until the rats were euthanized.

RU.521 (selective cGAS inhibitor) and 2′3′-cGAMP (STING agonist) were used in this study. Based on previous studies (Wang et al. 2021; Gamdzyk et al. 2020), RU.521 (#T5486, TargetMol, China, 450 μg/kg, dissolved in 1% DMSO + corn oil) or vehicle (1% DMSO diluted in corn oil) was intranasally administered at 2 h, 24 h, and 48 h after SBI, and 2′3′-cGAMP (#B8362, ApexBio Technology, USA, 500 μg/kg) or vehicle (saline) was injected intravenously through the tail vein 10 min before SBI, and this was repeated at 24 and 48 h after SBI. Furthermore, the groups used for neurological function assessment were treated for 7 days.

To assess the effective dose of vitamin C (#A4034, L-ascorbic acid, Sigma–Aldrich, USA), rats received intravenous administrations of low (100 mg/kg), medium (200 mg/kg) and high (500 mg/kg) doses of vitamin C one hour after SBI based on the previous literature (Chang et al. 2020). Vehicle-treated rats were injected intravenously with an equal volume of saline vehicle one hour after SBI. The procedures were repeated once a day until the animals were killed.

### Evaluation of Brain Water Content

The rats were anesthetized by the method described and euthanized by cervical dislocation. The fresh brains were quickly removed and separated into separate hemispheres. The right brain hemispheres were weighed immediately to determine the wet weight, and the samples were dried at 100 °C for 48 h to obtain the dry weight. Then the brain water content was determined as [(wet weight-dry weight)/wet weight] × 100%.

### Enzyme-Linked Immunosorbent Assay (ELISA)

Blood samples were collected using EDTA as an anticoagulant. Plasma was prepared by centrifugation at 1500 × g for 15 min at 4 °C. The plasma supernatant was removed, split into 0.5 mL aliquots, and assayed immediately or stored at − 80 °C until analysis. The levels of interleukin-6 (IL-6, #ml064292), tumor necrosis factor (TNF, #ml603709), Citrullinated histone H3 (CitH3, #ml002859), Myeloperoxidase (MPO)-DNA (#ml085663-J), interferon-β (IFN-β, #ml102842-J), and vitamin C (#ml003052) in plasma, brain tissue, or cell supernatant were measured using ELISA kits (Mlbio, Shanghai, China) according to the manufacturer’s guidelines. The absorbance at 450 nm was measured immediately with a multimode microplate reader (SpectraMax 190, Molecular Devices, USA).

### Neutrophil Isolation

Fresh whole blood was collected from the abdominal aorta of the rat and stored in EDTA anticoagulant tubes. Next, neutrophils were purified from the peripheral blood at room temperature by using a blood neutrophil isolation kit (#P9200, Solarbio, China) according to the manufacturer’s manual.

### Scanning Electron Microscopy

Neutrophils isolated from rat peripheral blood were seeded onto polylysine-coated coverslips. The coverslips and rat brain tissues were fixed with 2.5% glutaraldehyde for 24 h and with 1% osmium acid for 1 h. Then, they were dehydrated with a graded ethanol series in turn (30, 50, 70, 80, 90, and 100%). After drying, these specimens were coated with platinum and observed under scanning electron microscopy.

### Western Blot Analysis

Brain tissue samples were collected from the injured cortex and extracted with RIPA lysis buffer (#R0010, Solarbio, China). The protein concentration was determined by a BCA Protein Assay Kit (#PC0020, Solarbio, China). Protein samples (20 µg) were separated by SDS–PAGE and transferred to PVDF (#88518, Thermo Fisher Scientific, USA) membranes. Then the PVDF membranes were blocked in 5% skim milk for 2 h at room temperature and incubated overnight at 4 °C with the following primary antibodies: CitH3 (AB_304752, #ab5103, Abcam, 1:1000), GAPDH (AB_2107436, #60004-1-Ig, Proteintech, 1:50000), β-actin (AB_2687938, #66009-1-Ig, Proteintech, 1:20000), STING (AB_10665370, #19851-1-AP, Proteintech, 1:1000), β-Tubulin (AB_2881629, #66240-1-Ig, Proteintech, 1:20000), pTBK1 (AB_2840252, #AF8190, Affinity, 1:500), TBK1 (AB_2882504, #67211-1-Ig, Proteintech, 1:500). Then the membranes were incubated with an HRP-labeled secondary antibody at room temperature for 1 h. The protein bands were visualized using an ECL detection kit (#310209, ZETA LIFE, USA). All primary antibodies were validated and reported in published literature or by companies. ImageJ software was used to analyze the immunoblot images.

### Immunofluorescence

Neutrophils isolated from the peripheral blood of rats and primary rat microglia were seeded onto polylysine-coated coverslips in 24-well plates. After washing with PBS, the coverslips were fixed with 4% paraformaldehyde for 20 min. Then the coverslips were blocked with 1% BSA and incubated at 4 °C overnight with primary antibodies against CitH3 (AB_304752, #ab5103, Abcam, 1:1000), MPO (AB_2892996, #sc-390109, Santa Cruz, 1:100), Iba-1(AB_2820254, #17198, CST, 1:50) and IL-1β (AB_629741, #sc-52012, Santa Cruz, 1:100).

Rats were anesthetized deeply and transcardially perfused with ice-cold PBS followed by whole brains removed quickly, fixed in 4% paraformaldehyde overnight at 4 °C and dehydrated with 30% sucrose at 4 °C until sinking to the bottom. The brain samples were embedded in the optimal cutting temperature (O.C.T.) compound and stored at − 80 °C. The damaged cerebral hemispheres were then sliced into 8 µm-thick coronal sections using a freezing microtome (CM1950, Leica, Germany). The frozen sections were blocked for 1 h and incubated overnight at 4 °C with antibodies against CitH3 (AB_304752, #ab5103, Abcam, 1:1000), MPO (AB_2892996, #sc-390109, Santa Cruz, 1:100), Iba-1 (AB_667733, #sc-32725, Santa Cruz, 1:100), cGAS (AB_2770305, #A8335, ABclonal, 1:50), STING (AB_10665370, #19851-1-AP, Proteintech, 1:1000), and GFAP (AB_561049, #3670, CST, 1:400). Then the corresponding fluorescence-conjugated secondary antibodies were added and incubated for 2 h at room temperature. Nuclei were stained with 4’, 6-diamidino-2-phenylindole (DAPI, #ZLI-9557, ZSGB-BIO, China) for 10 min. All primary antibodies were validated in published literature or by companies. Finally, images of cells and brain sections were captured with laser confocal microscopy (LSM 800, Zeiss, Germany). We selected three sections per rat from similar areas which were around the injured frontal and parietal cortices, and analyzed three fields per section at a magnification of × 20 or × 40. Images were analyzed with ImageJ software. The relative immunofluorescence intensity of CitH3 or MPO was calculated by the ratio of immunofluorescence intensity of CitH3 or MPO relative to the immunofluorescence intensity of CitH3 or MPO in sham group respectively. The percentage of Iba-1-positive cells was calculated using the formula: (number of Iba-1-positive cells /number of total cells)*100, total cells were counted by DAPI. The positive cell results in each group were averaged.

### TUNEL Assay

A TUNEL kit (#C1088, Beyotime, China) was used to detect neuronal cell death according to the manufacturer’s protocol. Briefly, brain sections were incubated with NeuN (AB_2651140, #24307, CST, 1:50) at 4 °C overnight. Then, the sections were washed with PBS and incubated with TUNEL reaction mixture for 1 h at 37 °C, followed by staining with DAPI for 10 min. The TUNEL-positive neurons were observed by laser confocal microscopy. We selected three sections per rat. The average number of TUNEL-positive neurons was counted in four fields per section at a magnification of × 20, and quantified with the ImageJ software by the experimenter blinded to grouping. TUNEL-positive neurons were expressed as a percentage of the total neuron count.

### Neurological Function Evaluation

We used the modified Garcia test to assess neurological function on days 1, 3, and 7 after SBI, including seven evaluations: spontaneous activity, axial sensation, vibrissae touch, limb symmetry, lateral turning, forefoot movement, and climbing. The maximum score was 21 points, and each test received a score ranging from 0 (worst performance) to 3 (best performance). Details of the modified Garcia test are provided in the Supplementary Information. The evaluation was performed by two evaluators in a blinded manner.

### Primary Rat Microglia Culture and Coculture with Neutrophils

The primary rat microglia culture has been described in the previous literature (Bahrami et al. 2018). Briefly, 1–3-day-old neonatal SD rats were sterilized in 75% alcohol, and then cerebral cortices were dissociated and stripped of their blood vessels and meninges carefully under a dissecting microscope. After digestion of the clipped cerebral cortices using 0.25% trypsin-EDTA (#25200056, Gibco, USA), an equal volume of DMEM (#11965092, Gibco, USA) was added to terminate the digestion.

The single cell suspension was obtained by centrifugation and resuspension after filtration through a 75-µm mesh filter, and cultured in a 75 cm^2^culture flask at 37 °C in a 5% CO_2_ atmosphere. The culture medium was changed on days 3 and 7. After 12–14 days, primary rat microglia were obtained by shaking the mixed glial cultures at 200 rpm for 1 h. Then isolated microglia were treated in DMEM with 10% fetal bovine serum (#16140071, Gibco, USA), 1% penicillin/streptomycin (#15140122, Gibco, USA) and cultured in 5% CO_2_ at 37 °C.

Then, neutrophils isolated from rats in different groups were treated with DMEM-conditioned media (DCM) for 1 h as previously described (Zeng et al. 2022). Next, DCM-treated neutrophils (5 × 10^5^/well) were cocultured with primary rat microglia for 8 h using a Transwell coculture device (pore size 3 μm). Primary rat microglia were placed in the lower chambers embedded with cover slips, and neutrophils were localized in the upper chambers. Then microglia were observed under a confocal laser-scanning microscope, and the levels of inflammatory factors produced by activated microglia in the cell supernatant were measured by ELISA.

### Intracellular Reactive Oxygen Species (ROS) Measurement

Neutrophils were isolated from the peripheral blood of rats in different groups, including sham-operated, vehicle, and low-, medium-, and high-dose vitamin C-administered groups. Next, neutrophils (3 × 10^4^ cells/mL) were seeded into 96-well plates and loaded with 10 µM DCFH-DA (#S0033S, Beyotime, Shanghai, China) fluorescent probe at 37 °C for 30 min. After washing with PBS, the fluorescence intensity was measured with a multimode microplate reader (SpectraMax 190, Molecular Devices, USA).

### Live-cell Imaging

Purified neutrophils (1 × 10^5^ cells/mL) isolated from healthy rats were plated on a confocal dish and incubated with different concentrations of vitamin C (#A4034, Sigma–Aldrich, USA) for 30 min at 37 °C in a 5% CO_2_ atmosphere. Then the cells were stimulated with 100 nM PMA (#HY-18,739, MedChemExpress, USA) for 3 h at 37 °C and 5% CO_2_ atmosphere and imaged under a laser confocal microscopy (LSM 800, Zeiss, Germany). The cell-permeable fluorescent DNA dye Hoechst 33342 (#C0030, Solarbio, China) was mixed with cell-impermeable SYTOX Green (#S7020, Invitrogen, USA) to detect NETs.

### Statistical Analysis

All statistical analyses were performed with GraphPad Prism 9.0 (San Diego, CA, USA). All data are presented as the mean ± standard deviation (mean ± SD) or median (interquartile range). The normality of the data was assessed by Shapiro–Wilk test, and variance homogeneity was assessed by the F test and Brown–Forsythe test. The Mann–Whitney’s test and Kruskal–Wallis test were used to analyze differences for two groups and multiple groups respectively. Associations between variables were analyzed using Spearman correlation. A *P*-value < 0.05 was indicated as statistically significant.

## Results

### SBI Leads to the Formation of NETs in Circulating Blood and Damaged Brain Tissues

**Fig. 1:**
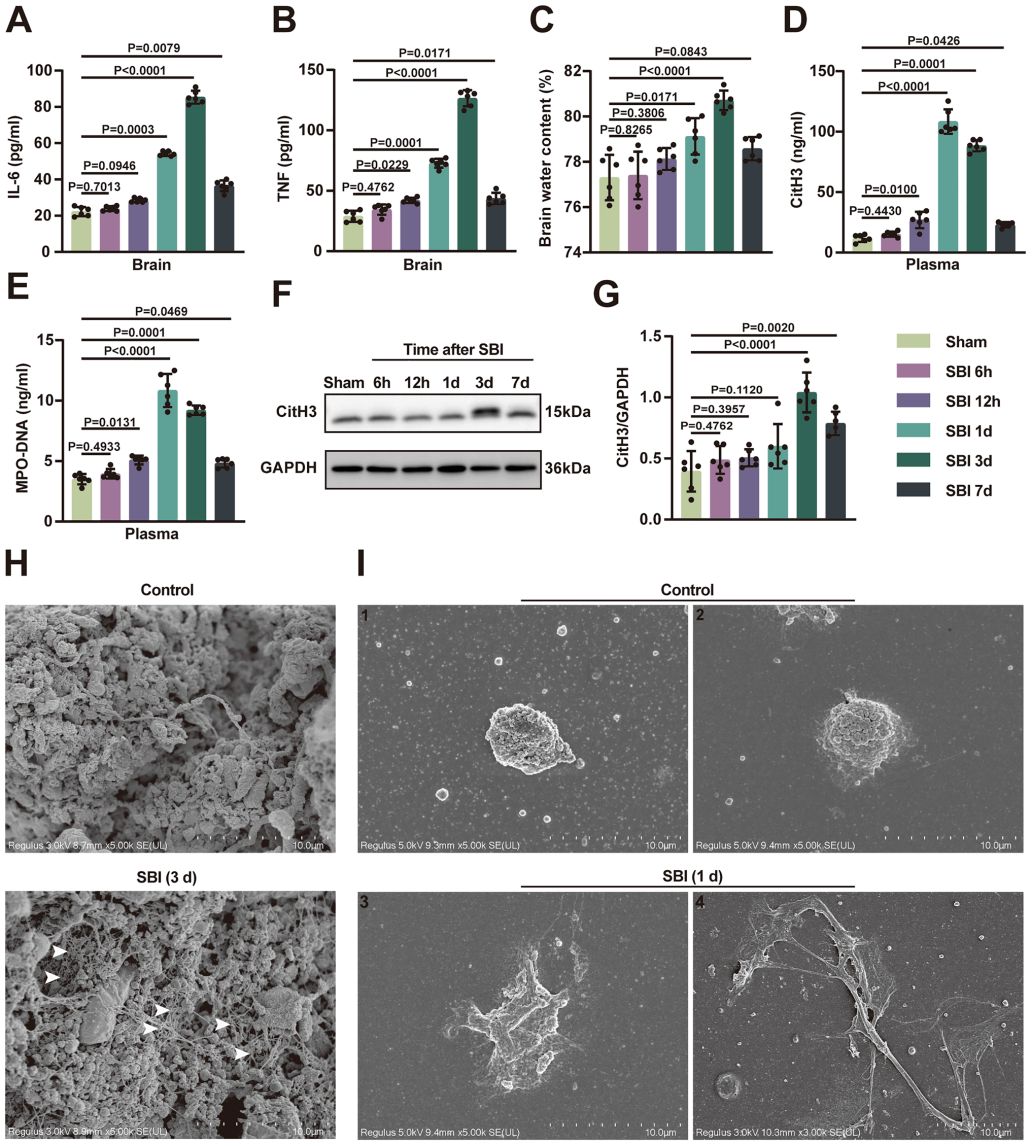
NETs were found in peripheral circulating blood and injured brain tissues after SBI. **A**,** B** Detection of inflammatory factor (IL-6, TNF) levels in rat brain tissues using ELISA at sham, 6 h, 12 h, 1 d, 3 d, and 7 d post-SBI (n = 6). **C** Brain water contents of the sham group and SBI group were measured at 1 d, 3 d, and 7 d post-SBI to assess brain edema (n = 6). **D**,** E** Detection of the levels of CitH3 and MPO-DNA, which are biomarkers of plasma NETs, using ELISA at sham, 6 h, 12 h, 1 d, 3 d, and 7 d post-SBI (n = 6). **F**,** G** Representative immunoblots (F) and quantification of CitH3 levels (G) in brain tissues of rats from the sham group and SBI group at 6 h, 12 h, 1 d, 3 d, and 7 d post-SBI (n =6). **H** Representative scanning electron micrographs of brain tissues in the control group and SBI 3d group, NET-like structures (white arrows) could be seen after SBI. Scale bar = 10 μm. **I** Neutrophils isolated from the control group (I1, I2) and SBI 1d group (I3, I4) were observed by scanning electron microscopy in vitro. Scale bar = 10 μm. The Kruskal–Wallis tests were used for statistical analysis. Data are presented as the mean ± SD. Details of statistical analysis are provided in the Supplementary Information

Neuroinflammation and brain edema are important factors in the development of SBI. We first detected neuroinflammation and cerebral edema at 6 h, 12 h, 1 day, 3 days, and 7 days after the onset of SBI. The expression levels of inflammatory factors were significantly elevated from 1d after SBI, peaked at 3 days and persisted for at least 7 days in the injured brain tissue (Fig. 1A and B). Similarly, brain edema was also most severe at 3 days in SBI rats compared to rats in the sham-operated group (Fig. 1C). To test whether NETs are present in SBI rats, we first detected the plasma levels of CitH3 and MPO-DNA, biomarkers of plasma NETs, and found that they were significantly elevated at 12 h in SBI rats, and then peaked at 1 day (Fig. 1D and E). We also found that the level of CitH3 in injured brain tissues of SBI rats increased in a time-dependent manner compared to that in sham-operated rats, reaching a peak level at 3 days (Fig. 1F and G). However, the time to peak of NETs was not the same in peripheral blood and brain tissue. Interestingly, the trends in the levels of NETs and inflammatory factors in brain tissue after SBI were broadly consistent, and both peaked on day 3.

Next, we observed extracellular NET-like structures adjacent to damaged brain parenchyma using scanning electron microscopy at 3 days after SBI. In contrast, no NETs were found in the brain tissue of control rats (Fig. 1H). We then isolated neutrophils from the peripheral blood of SBI and normal control rats and observed in vitro, using electron microscopy, that neutrophils isolated from SBI rats were more prone to form NETs or had formed NETs (Fig. 1I).

### Cl-Amidine and DNase I Treatment Prevent the NETs Formation

**Fig. 2:**
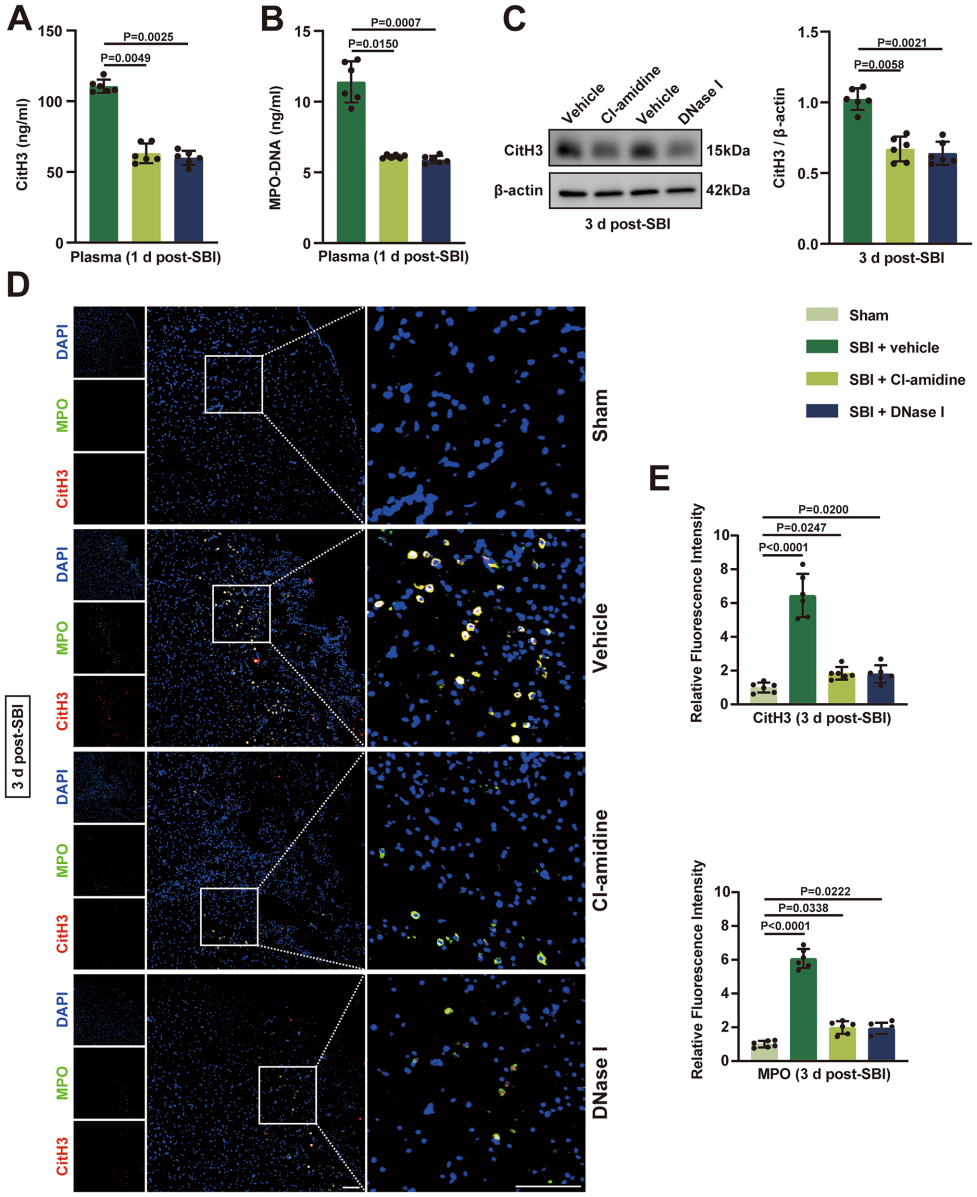
PAD4 inhibitor (Cl-amidine) and DNase I can significantly reduce the level of NETs after SBI. **A**,** B** Detection of plasma NETs levels in sham-operated and SBI rats after administration of Cl-amidine and DNase I using ELISA (n = 6). **C** Representative immunoblots and quantitative analyses of CitH3 in rat brain tissue after different interventions (n =6). **D** Representative immunofluorescence images of CitH3 (red) and MPO (green) double-positive cells in rat brain tissue from different groups at 3 days after SBI. Nuclei were stained with DAPI (blue). Scale bar = 100 μm. **E** Quantitative analyses of CitH3 and MPO in diverse groups (n = 6). The Kruskal–Wallis tests were used for statistical analysis. Data are presented as the mean ± SD. Details of statistical analysis are provided in the Supplementary Information

Peptidyl arginine deiminase 4 (PAD4) is critical for the formation of NETs (Thiam et al. 2020). DNase I, an endogenous NET-degrading enzyme, has been found to effectively disrupt NETs (Thiam et al. 2020). We investigated whether Cl-amidine (a pan-PAD inhibitor) and DNase I could effectively inhibit or degrade NETs post-SBI. The levels of NETs in peripheral blood, including plasma CitH3 and plasma MPO-DNA, were significantly reduced by administration of Cl-amidine or DNase I after SBI (Fig. 2A and B). In addition, we examined the levels of NETs in brain tissues of SBI rats after Cl-amidine and DNase I interventions using Western blot and immunofluorescence, respectively. In immunofluorescence staining of brain tissue, CitH3 and MPO co-staining represents the formation of NETs. We found that the level of NETs in damaged brain tissue was significantly reduced after administration of Cl-amidine, and degrading NETs with DNase I also had the same effect (Fig. 2C, D and E).

### Restricting NETs Plays a Neuroprotective Role After SBI

**Fig. 3:**
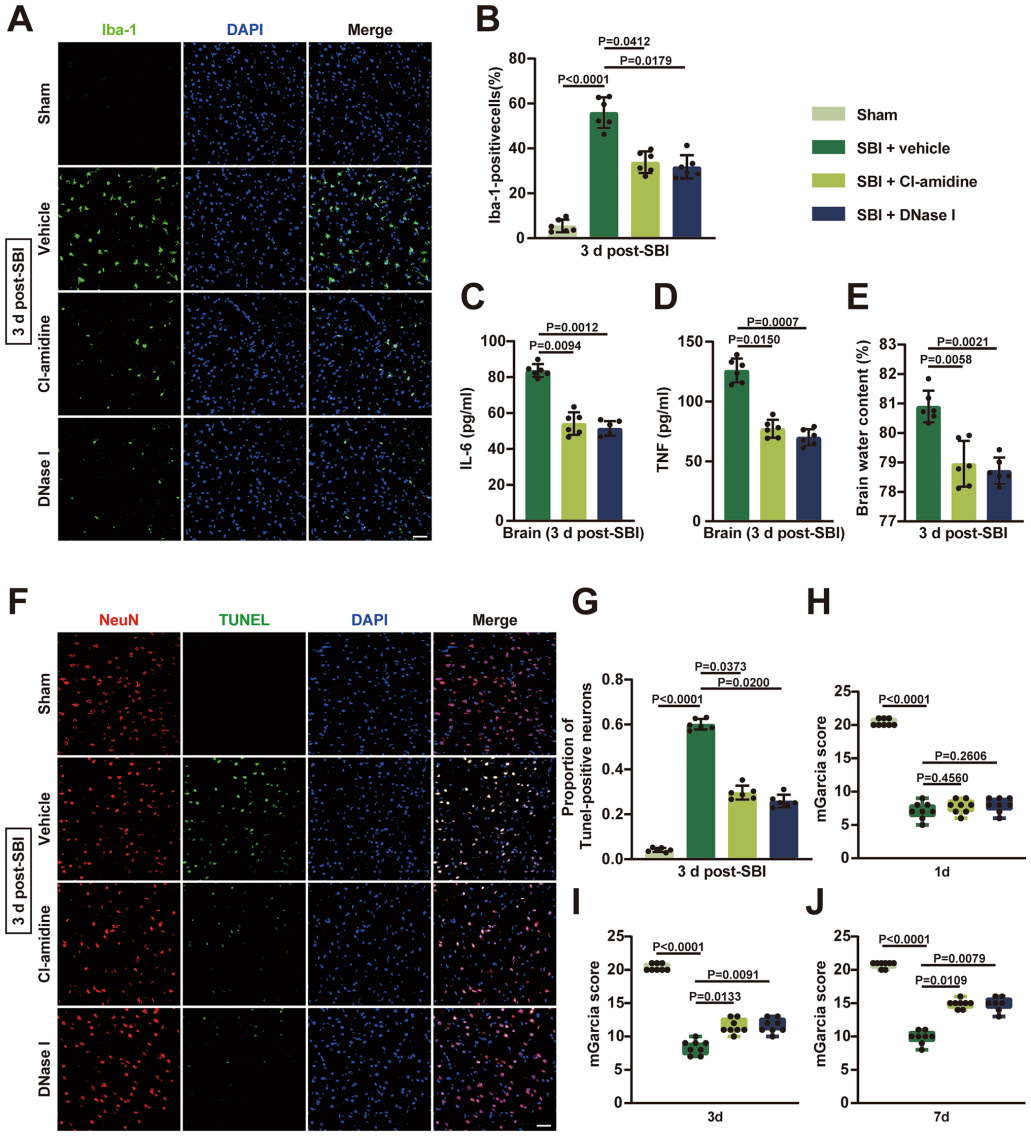
Restricting NETs attenuated neuroinflammation, and brain edema, alleviated neuronal cell death, and improved neurological function after SBI. **A** Representative immunofluorescence images of microglial activation in injured brain tissues of each group at 3 days after SBI (sham, SBI+vehicle, SBI+ Cl-amidine, SBI+ DNase I). Green, Iba-1; blue, DAPI; Scale bar = 50 μm. **B** Quantitative analyses of Iba-1 positive cells in each group (n = 6). **C**,** D** Detection of inflammatory factor (IL-6, TNF) levels in rat brain tissues of each group using ELISA at 3 days after SBI (n = 6). **E** Brain water contents were measured at 3 days post-SBI to assess brain edema (n = 6). **F** Representative immunofluorescence images of NeuN (neurons, red) and TUNEL (green) in rat brain tissues of each group at 3 days after SBI. Scale bar = 50 μm. **G** Quantification of the percentage of TUNEL-positive neurons (n = 6). **H**,** I**,** J** The modified Garcia scores were used to assess neurological deficits in each group at 1 day, 3 days, and 7 days (n = 8). The Kruskal–Wallis tests were used for statistical analysis. Data are presented as the mean ± SD (B, C, D, E, and G) or median (interquartile range) (**H**, **I**, and **J**). Details of statistical analysis are provided in the Supplementary Information

Increasing evidence indicates that excess formation of NETs is often unfavorable in acute neurological disorders (Vaibhav et al. 2020; Zeng et al. 2022; Wang et al. 2021). We explored the role of NETs and whether inhibiting NETs formation is beneficial post-SBI. Microglia play an important role in the development of neuroinflammation after brain injury. Immunofluorescence staining showed that expression of Iba-1 protein in the surrounding peri-resection brain tissues of SBI rats significantly increased compared with that in the sham-operated rats, and treatment with Cl-amidine and DNase I noticeably reduced microglial activation (Fig. 3A and B). Cl-amidine or DNase I administration reduced the levels of inflammatory factors (IL-6, TNF) and the water content of brain tissue compared with the SBI + vehicle group on the 3rd-day post-SBI (Fig. 3C, D and E).

Furthermore, TUNEL staining results showed that the number of TUNEL-positive neurons were increased in the injured brain tissues of the SBI + vehicle group compared with the sham group, whereas administration of Cl-amidine or DNase I significantly decreased the number of dead neurons (Fig. 3F and G). The modified Garcia scores were used to assess neurological function. We found that the neurological deficit in each group was most severe at 1 day and recovered over time (Fig. 3H). At 3 days and 7 days, the neurological function scores were considerably improved in the Cl-amidine and DNase I groups compared with the SBI and vehicle groups (Fig. 3I and J). Our results suggest that the formation of NETs in SBI may be detrimental, and restricting NETs attenuated neuroinflammation, and brain edema, alleviated neuronal cell death, and improved neurological function post-SBI.

### The cGAS-STING Pathway is Triggered by SBI

**Fig. 4:**
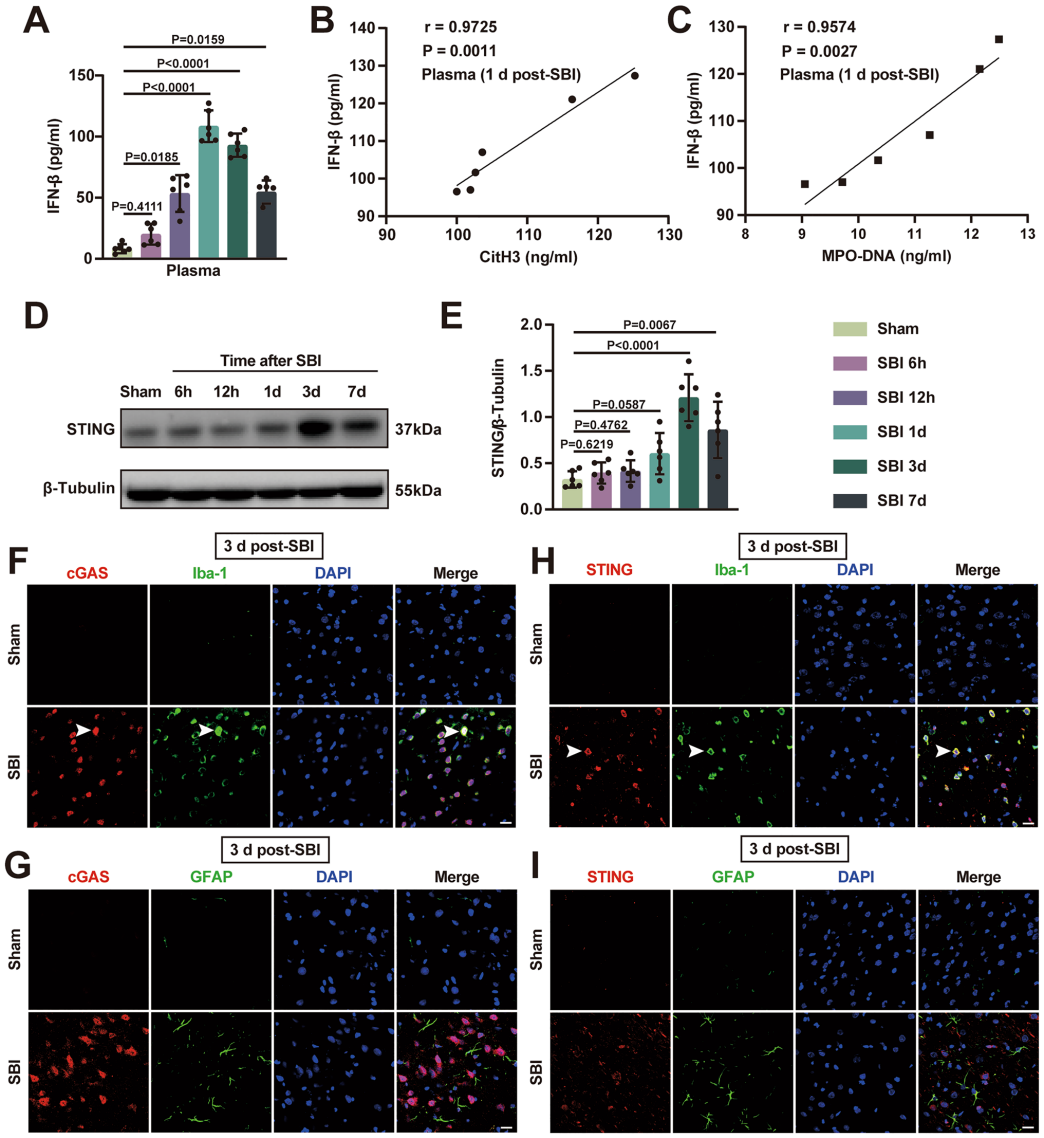
The cGAS-STING pathway is activated after SBI. **A** Quantification of the levels of plasma IFN-β using ELISA at sham, 6 h, 12 h, 1 d, 3 d, and 7 d post-SBI (n = 6). **B** Plasma CitH3 correlated with plasma IFN-β, r = 0.9725, P = 0.0011 (n = 6). **C** Plasma MPO-DNA correlated with plasma IFN-β, r = 0.9574, P = 0.0027 (n = 6). **D**,** E** Representative immunoblots and quantitative analyses of STING expression in injured brain tissues of rats from the sham group and SBI group at 3 days post-modeling (n = 6). **F–I** Representative images of immunofluorescence double staining showing the localization of cGAS (red) and STING (red) with Iba-1 (green) and GFAP(green) respectively, in the brain tissues of sham-operated and SBI rats. Scale bar = 20 μm. The Kruskal–Wallis tests were used for statistical analysis (**A**, **E**). Spearman correlation was used to analyze the correlation between the levels of plasma CitH3, plasma MPO-DNA and the level of IFN-β in SBI rats (**B**, **C**). Data are presented as the mean ± SD. Details of statistical analysis are provided in the Supplementary Information

The plasma IFN-β levels were significantly elevated from 12 h after SBI, peaked at 1 day and persisted for at least 7 days (Fig. 4A). The trend of plasma IFN levels was generally consistent with that of circulating NETs levels after SBI. Interestingly, circulating NETs levels (plasma CitH3 and MPO-DNA) positively correlated with the levels of plasma IFN-β on day 1 in SBI rats (Fig. 4B and C), suggesting that NETs may be associated with the cGAS-STING pathway after SBI. Subsequently, we detected significantly increased levels of STING in injured brain tissues of SBI rats, and the protein expression of STING peaked at 3 days in rats subjected to SBI (Fig. 4D and E). Furthermore, immunofluorescence staining confirmed that cGAS and STING were mainly distributed in activated microglia (as indicated by the white arrows) rather than astrocytes at 3 days after SBI (Fig. 4F).

### Blocking the cGAS-STING Pathway is Neuroprotective After SBI

**Fig. 5:**
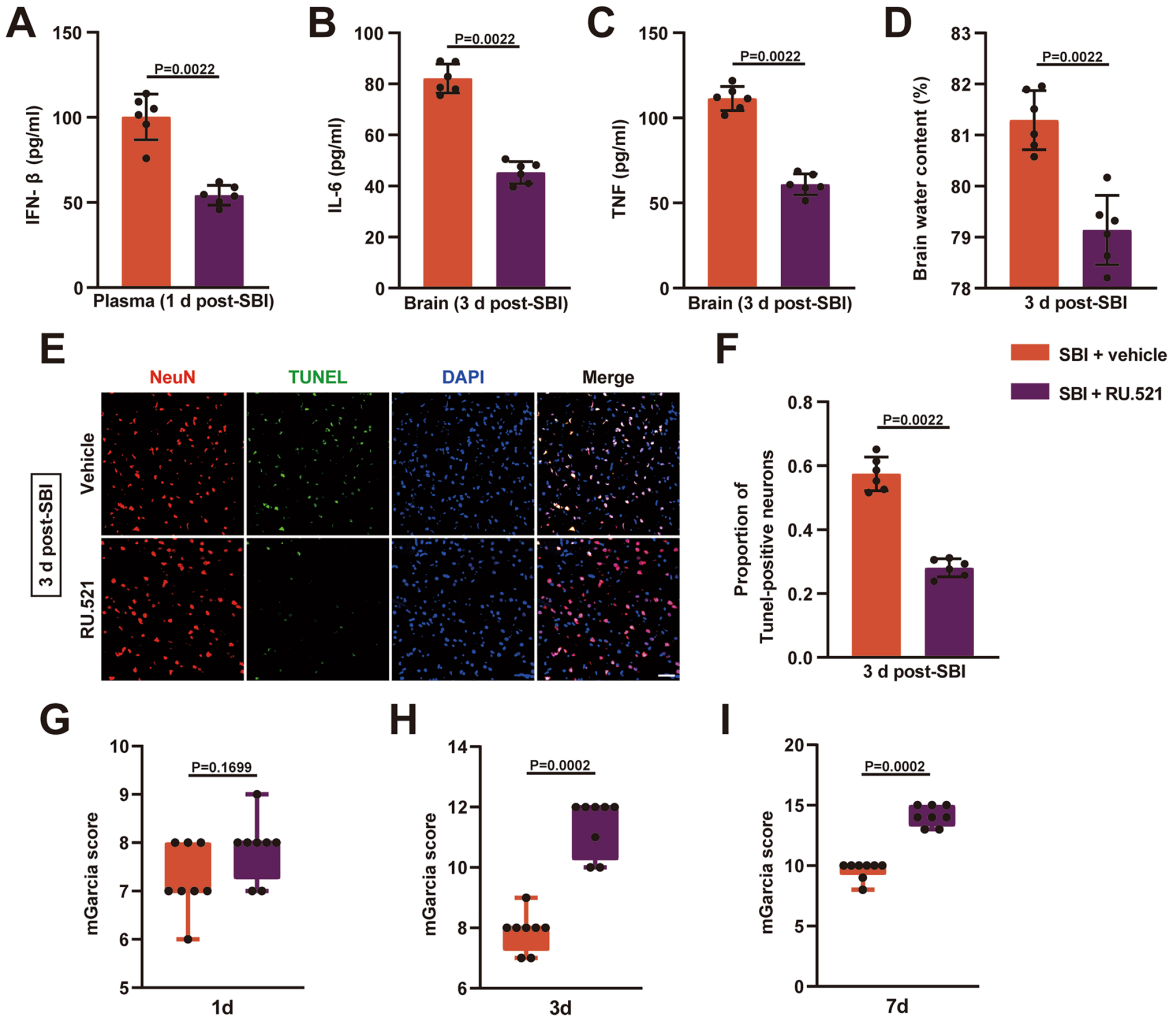
Blockade of cGAS-STING using RU.521 can be neuroprotective after SBI. **A** Quantification of the levels of plasma IFN-β using ELISA in the SBI + vehicle group and SBI + RU.521 group on day 1 post-SBI (n = 6). **B**,** C** Detection of inflammatory factor (IL-6, TNF) levels in rat brain tissues of the two groups using ELISA at 3 days after SBI (n = 6). **D** Quantification of brain water content at 3 days after SBI (n = 6). **E** Representative immunofluorescence images of NeuN (red) and TUNEL (green) in the two groups at 3 days after SBI. **F** Quantitative analysis of the percentage of TUNEL-positive neurons (n = 6). **G–I** Quantification of neurological function using modified Garcia scores at 1 day, 3 days, and 7 days post-SBI (n = 8). Mann–Whitney’s tests were used for statistical analysis. Data are presented as the mean ± SD (**A**, **B**, **C**, **D**, and **F**) or median (interquartile range) (**G**, **H**, and **I**). Details of statistical analysis are provided in the Supplementary Information

We used RU.521, a specific inhibitor of cGAS, to investigate the role of cGAS-STING in the pathological process after SBI. RU.521 significantly reduced IFN levels in plasma 1 day after SBI (Fig. 5A). Compared with the SBI + vehicle group, rats in the SBI + RU.521 group demonstrated markedly decreased inflammatory factors (IL-6, TNF) in impaired brain tissues at 3 days after SBI (Fig. 5B and C). Similarly, the brain water content in the SBI + vehicle group was significantly lower (Fig. 5D). Furthermore, we found that treatment with RU.521 significantly inhibited neuronal cell death (Fig. 5E and F) and attenuated neurological impairments at 3 days and 7 days after SBI (Fig. 5H and I). However, the neurologic function scores did not differ between the two groups on day 1 (Fig. 5G).

### NETs are Important for SBI-Induced Activation of the cGAS-STING Pathway

**Fig. 6:**
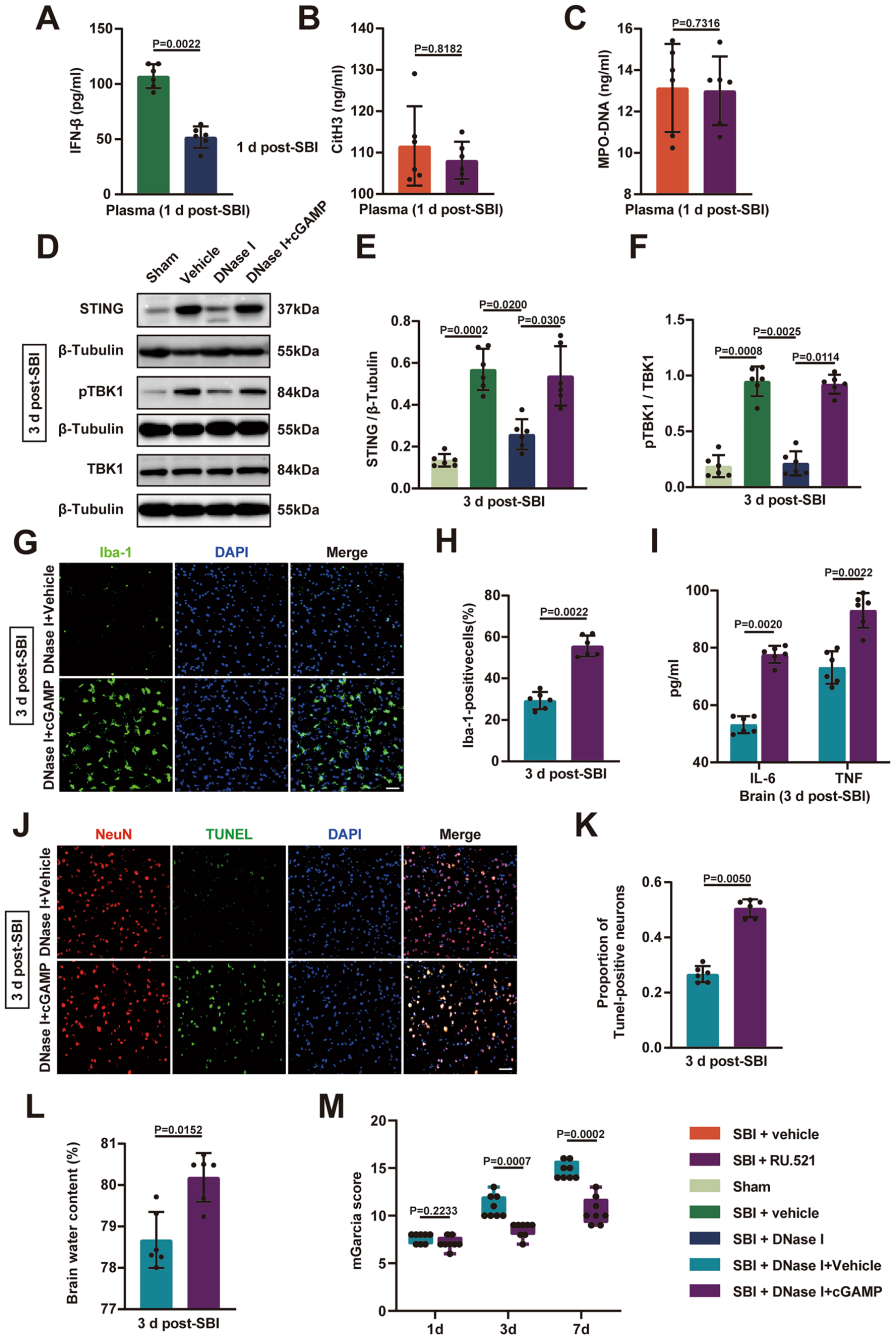
Degradation of NETs with DNase I inhibits the cGAS-STING pathway after SBI.**A** Quantification of the levels of plasma IFN-β using ELISA at 1 day in SBI rats treated with vehicle, and DNase I (n = 6). **B**,** C** Quantification of the levels of plasma CitH3 and plasma MPO-DNA using ELISA at 1 day in SBI rats treated with vehicle, and RU.521 (n = 6). **D–F** Representative immunoblots and quantification of STING, phosphorylated (pTBK1), and total TBK1 expression in the injured brain tissues at 3 days in sham-operated rats and SBI rats treated with vehicle, DNase I, and DNase I in combination with cGAMP (n = 6). **G**,** H** Representative immunofluorescence images of microglia (Iba-1, Green) activation in injured brain tissues of the two groups at 3 days post-SBI (DNase I + vehicle, DNase I + cGAMP). Scale bar = 50 μm. **I** Detection of inflammatory factor (IL-6, TNF) levels in rat brain tissues of the two groups using ELISA at 3 days (n = 6). **J** Representative immunofluorescence images of NeuN (red) and TUNEL (green) in rat brain tissues of the two groups at 3 days post-SBI (DNase I + vehicle, DNase I + cGAMP). Scale bar = 50 μm. **K** Quantification of the percentage of TUNEL-positive neurons (n = 6).**L** Brain water contents were measured at 3 days to assess brain edema in the two groups (n = 6). **M** The modified Garcia scores were used to assess neurological deficits in the two groups at 1 day, 3 days, and 7 days after SBI (n = 8).Statistical analysis was performed using Mann–Whitney’s test (**A**, **B**, **C**, **H**, **I**, **K**, **L**, and **M**), and Kruskal–Wallis test (**E**, **F**). Data are presented as the mean ± SD (**A**, **B**, **C**, **E**, **F**, **H**, **I**, **K** and **L**) or median (interquartile range) (**M**). Details of statistical analysis are provided in the Supplementary Information

SBI triggered activation of the cGAS-STING pathway. DNase I treatment significantly inhibited SBI-induced upregulation of STING-related IFN-β in plasma at 1 day (Fig. 6A). However, blocking the cGAS-STING pathway with RU.521 did not result in changes in the levels of circulating NETs (plasma CitH3, plasma MPO-DNA) after SBI (Fig. 6B and C). We found that the protein levels of STING and TANK-binding kinase 1 (TBK1) increased in the injured brain tissues of rats subjected to SBI at 3 days, whereas this SBI-induced activation of STING and TBK1 was abolished by DNase I (Fig. 6D–F). The decrease in SBI-induced activation of the cGAS-STING pathway caused by DNase I was reversed by combination with the cGAS product cyclic GMP-AMP (cGAMP) (Fig. 6D–F).

Next, we found that inhibition of microglial activation (Fig. 6G and H) and suppression of proinflammatory factor (IL-6, TNF) production (Fig. 6I) in rat brain tissue by DNase I was abolished by cGAMP treatment at 3 days after SBI. Consistent with the increase in the number of dead neurons (Fig. 6J and K), infusion of cGAMP reversed the effect of reduction of cerebral edema (Fig. 6L) with DNase I treatment and exacerbated neurological impairment in SBI rats at 3 d and 7 d (Fig. 6M). The results showed that the degradation of NETs by DNase I inhibited the cGAS-STING pathway after SBI. Similarly, NETs participated in the SBI damage process via the cGAS-STING signaling pathway.

### cGAMP Reverses the Effect of DNase I on SBI-Induced Neuroinflammation In Vitro

**Fig. 7:**
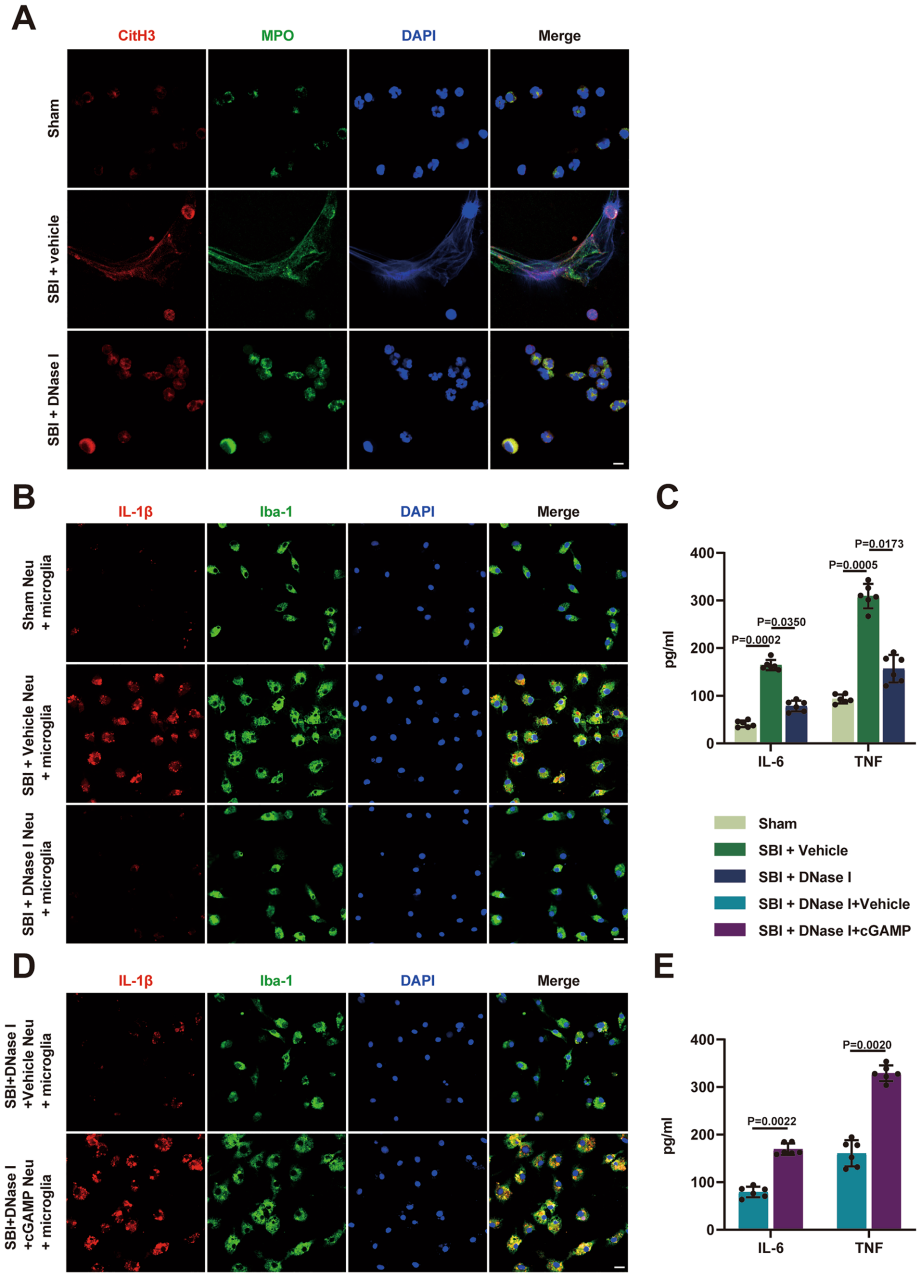
DNase I-mediated attenuation of neuroinflammation is abolished by cGAMP in vitro. **A** Representative immunofluorescence images of isolated peripheral blood neutrophils from rats undergoing sham surgery and SBI rats treated with vehicle or DNase I. Neutrophils were stained with CitH3 (red) and MPO (green). Scale bar = 5 μm. **B** Representative pictures of primary rat microglia cocultured with neutrophils isolated from sham-operated rats, and SBI rats treated with vehicle or DNase I. Microglia were stained with IL-1β (red) and Iba-1 (green). Neu = neutrophils, Scale bar = 20 μm. **C** Detection of inflammatory factor (IL-6, TNF) levels in cocultured microglia supernatants in different groups (n = 6). **D** Representative pictures of primary rat microglia cocultured with neutrophils in two groups with different treatments. Microglia were stained with IL-1β (red) and Iba-1 (green). Neu = neutrophils, Scale bar = 20 μm. **E** Detection of inflammatory factor (IL-6, TNF) levels in cocultured microglia supernatants in different groups (n = 6). Statistical analysis was performed using Kruskal–Wallis test (**C**) and Mann–Whitney’s test (**E**). Data are presented as the mean ± SD. Details of statistical analysis are provided in the Supplementary Information

We demonstrated that the formation of NETs after SBI can activate the cGAS-STING pathway in vivo, further activate microglia, and exacerbate neuroinflammation. Using immunofluorescence, we found that there was no NETs formation in neutrophils from the sham-treated rats, neutrophils from vehicle-treated rats showed NETs formation, and DNase I treatment degraded NETs (Fig. 7A). We then explored the effect of NETs on microglia in vitro. Primary rat microglia were cocultured with neutrophils isolated from sham-treated, vehicle-treated, and DNase I-treated rats. After coculturing, the morphological changes in the primary rat microglia were obvious. Primary rat microglia cocultured with neutrophils from vehicle-treated rats were more likely to become activated (Fig. 7B). Immunofluorescence staining showed that the expression of IL-1β was increased in primary rat microglia cocultured with neutrophils from vehicle-treated rats, whereas, DNase I-treated neutrophils reversed this change in microglia (Fig. 7B). The levels of pro-inflammatory factors (IL-6, TNF) in the supernatants of primary rat microglia were significantly elevated in the SBI + vehicle group, while the upregulation of pro-inflammatory factors was significantly diminished in the SBI + DNase I group (Fig. 7C).

We further investigated whether DNase I attenuates microglia-mediated neuroinflammation via the cGAS-STING pathway in vitro. Before microglia were cocultured with neutrophils from DNase I-treated SBI rats, the vehicle (phosphate-buffered saline, PBS) and cGAS product cGAMP (20 µg/mL) were added to the Transwell coculture device for 30 min. We then found that cGAMP reversed this neuroprotective effect of DNase I, including the morphological changes and the expression of IL-1β in microglia, and proinflammatory factor levels in the supernatant (Fig. 7D and E).

### Intervention with High-Dose Vitamin C Inhibits the Formation of NETs After SBI

**Fig. 8:**
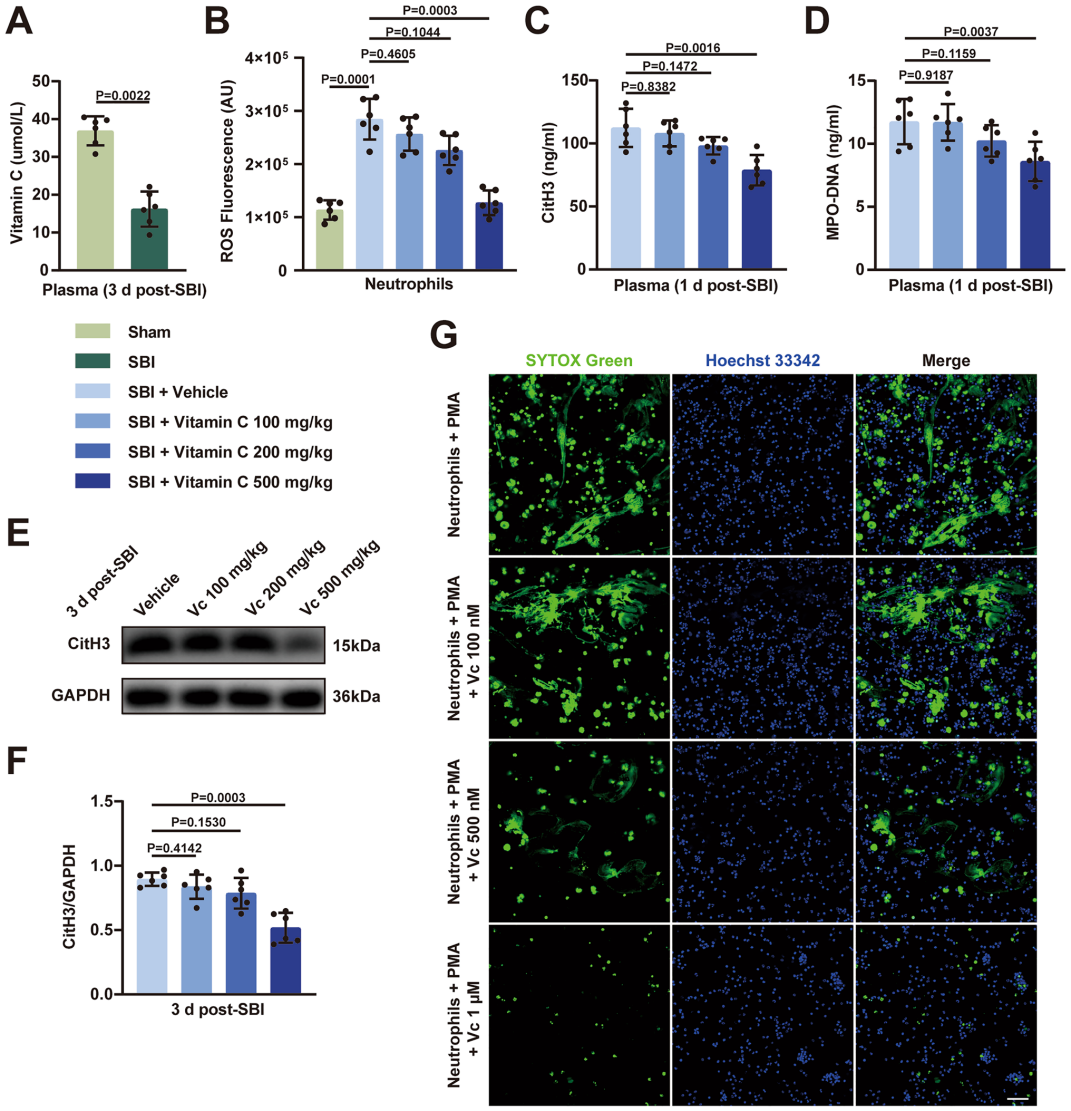
Effect of vitamin C treatment on the release of NETs after SBI. **A** Detection of vitamin C levels in the plasma of SBI rats and sham-operated rats using ELISA at 3 days post-modeling (n = 6). **B** The ROS levels of neutrophils isolated from peripheral blood were measured at 1 day post-SBI in different intervention groups, including a sham group, a vehicle group, and SBI rats that received low (100 mg/kg), medium (200 mg/kg), or high doses (500 mg/kg) of vitamin C (n = 6). **C**,** D** Quantification of the levels of plasma CitH3 and plasma MPO-DNA in the four groups of rats at 1 day after SBI (n = 6). **E**,** F** Representative immunoblots and quantitative analyses of CitH3 in brain tissue after interventions with different doses of vitamin C at 3 days after SBI (n = 6). **G** Typical images of live-cell-forming NETs can be visualized by extracellular DNA (SYTOX Green) and intracellular DNA (Hoechst 33342) on laser confocal microscopy. Scale bar = 50 µm. Statistical analysis was performed using Mann–Whitney’s test (**A**) and Kruskal–Wallis test (**B**, **C**, **D**, and **F**). Data are presented as the mean ± SD. Details of statistical analysis are provided in the Supplementary Information

We first explored whether plasma levels of vitamin C were altered after SBI. We found that plasma levels of vitamin C in the SBI group were significantly decreased compared with those in the sham-operated group, indicating that plasma levels of vitamin C could be affected after SBI (Fig. 8A). Based on the fact that ROS is a key factor in the formation of NETs, we examined the effects of different doses of vitamin C administered intravenously on ROS generation in neutrophils in the circulating blood of SBI rats. We found that only high-dose (500 mg/kg) vitamin C reduced neutrophil ROS production after intravenous injection (Fig. 8B).

Next, we investigated whether vitamin C administration could interfere with the formation of NETs. We examined the levels of NETs in the plasma and brain tissue of rats in different vitamin C dose groups using ELISA and western blot respectively. We found that only high-dose vitamin C effectively inhibited the formation of NETs in the circulating blood (Fig. 8C and D) and brain tissues around the injured foci (Fig. 8E and F) after SBI, and neither low nor medium doses reduced the formation of NETs. We further confirmed the effect of different concentrations of vitamin C on the formation of NETs in vitro. PMA was used as a positive control. Different concentrations of vitamin C were cocultured with neutrophils prior to PMA stimulation, and other culture conditions in each group were kept the same as those in the positive control group. We visualized NETs using Hoechst 33342 and SYTOX Green and found that only a high concentration of 1 μm vitamin C significantly reduced NETs using live-cell imaging. However, 500 nM only slightly reduced NETs, and 100 nM had almost no effect (Fig. 8G).

## Discussion

SBI has largely been overlooked. Currently, the pathophysiological mechanisms have yet to be fully defined, and there is no effective treatment to protect the brain from SBI (Eser Ocak et al. 2020). To our knowledge, this is the first study to investigate the role of NETs in the pathophysiological process following SBI and explore the relevant mechanisms. In the present study, we found that SBI could lead to the formation of NETs, which promoted neuroinflammation, brain edema, and neuronal cell death, and aggravated neurological deficits through the cGAS-STING pathway. SBI-induced damage could be attenuated by clearing NETs with DNase I or inhibiting NETs formation with Cl-amidine. Furthermore, we demonstrated that high-dose vitamin C administration significantly inhibits the formation of NETs after SBI, and it could be a promising therapy that is closer to clinical translation.

A growing number of studies have found that NETs play an important role in CNS disorders and that excessive formation of NETs is often harmful to patients (Denorme et al. 2022; Li et al. 2022). Neutrophils cannot enter the undamaged central nervous system due to the blood–brain barrier (BBB); however, the impaired BBB permits neutrophils to infiltrate into brain tissues following injury (Vaibhav et al. 2020). We showed that the levels of NETs in peripheral blood peaked at 1 d after SBI, while NETs in damaged brain tissue peaked at 3 d, which was partially consistent with previous studies (Kim et al. 2019; Kang et al. 2020). The temporal and spatial progress of NETs formation post-SBI needs further exploration. NET-like structures were found in the brain tissues of SBI rats by using scanning electron microscopy, and neutrophils isolated from SBI rats tended to form spontaneous NETs. We found that either inhibition of NETs with Cl-amidine or degradation of NETs with DNase I could attenuate neuroinflammation, cerebral edema, and neuronal cell death, and improve neurological function after SBI. This suggests that SBI-induced excessive formation of NETs is deleterious and that inhibition of NETs formation can be neuroprotective.

The cGAS-STING pathway is closely associated with neuroinflammation, which has been demonstrated in acute brain injuries, such as traumatic brain injury (Abdullah et al. 2018; Barrett et al. 2021), subarachnoid hemorrhage (Peng et al. 2020), and ischemic stroke (Liao et al. 2020). The plasma levels of IFN-β were significantly elevated post-SBI, and circulating NETs levels positively correlated with plasma IFN-β levels. These results indicate that NETs may be associated with the cGAS-STING pathway after SBI. Furthermore, the levels of STING in brain tissues were significantly elevated on day 3 after SBI. Notably, elevated cGAS and STING were mainly localized in microglia rather than astrocytes after SBI. These findings suggest that microglia-mediated inflammation may be triggered by the activation of the cGAS-STING pathway (Liao et al. 2020). We also found that blocking cGAS/STING activation with RU.521 significantly alleviated SBI-induced injuries, including neuroinflammation, brain edema, neuronal cell death, and neurological deficits.

Next, we further explored the relationship between NETs and the cGAS-STING pathway after SBI. We found that DNase I markedly suppressed the activation of the cGAS-STING pathway, which was reversed by the cGAS product cGAMP. In addition, the neuroprotective effect of DNase I in SBI was also abolished by cGAMP. The results demonstrated that NETs participated in the pathological progression of SBI through the cGAS-STING signaling pathway. It has been proposed that NETs can induce neuronal death in vitro (Kim et al. 2019). Based on previous research methods (Zeng et al. 2022; Kim et al. 2019), we found that primary rat microglia cocultured with neutrophils from vehicle-treated SBI rats were more likely to become activated, and proinflammatory factors released by microglia significantly increased. DNase I-treated neutrophils reversed this change in microglia. Furthermore, DNase I attenuated microglia-mediated neuroinflammation via the cGAS-STING pathway in vitro. Our data revealed that NETs released from neutrophils promote microglia-mediated neuroinflammation via the cGAS-STING pathway in vitro.

Our data suggest that targeting NETs or cGAS-STING can be neuroprotective after SBI. To make this therapeutic strategy more readily applicable to the clinic, we further explored whether vitamin C could inhibit the formation of NETs after SBI. Vitamin C, as an extremely critical antioxidant molecule in the brain, plays an important role in the normal function of the central nervous system (Kocot et al. 2017). A growing number of studies have demonstrated a significant link between vitamin C deficiency and brain injury, and exogenous administration of vitamin C may be beneficial (Sanchez-Moreno et al. 2004; Leichtle et al. 2020; Kangisser et al. 2021). A study showed that only intravenous administration of vitamin C produces high plasma concentrations that might have therapeutic activity (Padayatty et al. 2004). Here, we found that plasma levels of vitamin C significantly decreased after SBI. The production of ROS is a key factor in the formation of NETs (Papayannopoulos 2018). High-dose vitamin C reduced neutrophil ROS production after intravenous injection. We then found that only high-dose vitamin C could effectively inhibit the formation of NETs in the circulating blood and brain tissues after SBI. Moreover, the same effect of a high concentration of vitamin C was validated in vitro. These results suggest that only high doses of vitamin C can effectively inhibit the formation of NETs, and may offer a new approach for the treatment of SBI. Currently, little attention has been paid to the application of anti-NETs therapies to surgical practice. The administration of recombinant human DNase (rhDNase) to inhibit NETs is a potential approach for postoperative treatment (Eustache et al. 2020). The use of anti-NETs therapies for SBI to improve surgical outcome requires more in-depth investigation.

Nevertheless, there were several limitations in this study. Firstly, the sample size of the experiments was not precalculated and was determined according to previous experience (Liao et al. 2023). Only male rats were used in our study, gender differences in SBI outcomes should be evaluated in future studies. Secondly, the underlying mechanisms of SBI-induced NETs formation need further investigation. Thirdly, DNase I can degrade various forms of DNA and it does not just specifically target NETs. Although DNase I treatment reduced cGAS-STING after SBI, this effect might not be solely due to NET degradation. The cGAS-STING pathway needs to be validated using knockout animals in future studies. In addition, it remains unclear the mechanisms by which high doses of vitamin C inhibit NETs formation. The clinical feasibility of vitamin C therapy requires more studies in the future.

In conclusion, our findings demonstrate that NETs might be involved in the pathogenesis of SBI by acting through the cGAS-STING pathway. We suggest that targeting NETs is a novel therapeutic strategy for SBI treatment, and high-dose vitamin C intervention may be a promising translational therapy for patients.

## Supplementary Information

Below is the link to the electronic supplementary material.

Supplementary material 1 (PDF 830.4 kb)

## Data Availability

The data and materials that support the findings of this study are available from the corresponding author upon reasonable request.

## Abbreviations

NETs: Neutrophil extracellular traps
SBI: Surgical brain injury
cGAMP: Cyclic guanosine monophosphate–adenosine monophosphate
cGAS: Cyclic guanosine monophosphate–adenosine monophosphate synthase
STING: Synthase-stimulator of interferon genes
PAD: Peptidylarginine deiminase
DNase I: Deoxyribonuclease I
CNS: Central nervous system
IFN: Interferon
ELISA: Enzyme-linked immunosorbent assay
TUNEL: Terminal deoxynucleotidyl transferase dUTP nick-end labeling
IL-6: Interleukin-6
TNF: Tumor necrosis factor
CitH3: Citrullinated histone H3
MPO: Myeloperoxidase
ROS: Reactive oxygen species
PMA: Phorbol 12-myristate 13-acetate
TBK1: TANK-binding kinase 1
