# Supplementary material for: Neutrophil Extracellular Traps Regulate Surgical Brain Injury by Activating the cGAS-STING Pathway
Source: Cell Mol Neurobiol. 2024 Apr 18;44:36. doi: 10.1007/s10571-024-01470-9 (PMC11026279; doi:10.1007/s10571-024-01470-9)
Supplement: Supplementary file 1 — Supplementary material 1 (PDF 830.4 kb) [file 10571_2024_1470_MOESM1_ESM.pdf]

## Normal distribution detection (Except for the red-marked data, all data conform to a normal distribution)

### Figure 1A

| Normality and Lognormality Tests<br>Tabular results | A | B | C | D | E | F |
|---|---|---|---|---|---|---|
| | Sham | SBI 6h | SBI 12h | SBI 1d | SBI 3d | SBI 7d |
| Test for normal distribution | | | | | | |
| Shapiro-Wilk test | | | | | | |
| W | 0.9095 | 0.8796 | 0.8355 | 0.8500 | 0.9574 | 0.9485 |
| P value | 0.4328 | 0.2671 | 0.1196 | 0.1573 | 0.7993 | 0.7280 |
| Passed normality test (alpha=0.05)' | Yes | Yes | Yes | Yes | Yes | Yes |
| P value summary | ns | ns | ns | ns | ns | ns |

### Figure 1B

| Normality and Lognormality Tests<br>Tabular results | A | B | C | D | E | F |
|---|---|---|---|---|---|---|
| | Sham | SBI 6h | SBI 12h | SBI 1d | SBI 3d | SBI 7d |
| Test for normal distribution | | | | | | |
| Shapiro-Wilk test | | | | | | |
| W | 0.8836 | 0.8133 | 0.9737 | 0.9429 | 0.8680 | 0.9079 |
| P value | 0.2859 | 0.0772 | 0.9161 | 0.6823 | 0.2183 | 0.4227 |
| Passed normality test (alpha=0.05)' | Yes | Yes | Yes | Yes | Yes | Yes |
| P value summary | ns | ns | ns | ns | ns | ns |

### Figure 1C

| Normality and Lognormality Tests<br>Tabular results | A | B | C | D | E | F |
|---|---|---|---|---|---|---|
| | Sham | SBI 6h | SBI 12h | SBI 1d | SBI 3d | SBI 7d |
| Test for normal distribution | | | | | | |
| Shapiro-Wilk test | | | | | | |
| W | 0.9309 | 0.9398 | 0.9543 | 0.9348 | 0.9839 | 0.8221 |
| P value | 0.5869 | 0.6578 | 0.7749 | 0.6179 | 0.9692 | 0.0921 |
| Passed normality test (alpha=0.05)' | Yes | Yes | Yes | Yes | Yes | Yes |
| P value summary | ns | ns | ns | ns | ns | ns |

### Figure 1D

| Normality and Lognormality Tests<br>Tabular results | A | B | C | D | E | F |
|---|---|---|---|---|---|---|
| | Sham | SBI 6h | SBI 12h | SBI 1d | SBI 3d | SBI 7d |
| Test for normal distribution | | | | | | |
| Shapiro-Wilk test | | | | | | |
| W | 0.9308 | 0.9871 | 0.9210 | 0.8052 | 0.9365 | 0.8975 |
| P value | 0.5866 | 0.9810 | 0.5125 | 0.0654 | 0.6310 | 0.3593 |
| Passed normality test (alpha=0.05)' | Yes | Yes | Yes | Yes | Yes | Yes |
| P value summary | ns | ns | ns | ns | ns | ns |

### Figure 1E

| Normality and Lognormality Tests<br>Tabular results | A | B | C | D | E | F |
|---|---|---|---|---|---|---|
| | Sham | SBI 6h | SBI 12h | SBI 1d | SBI 3d | SBI 7d |
| Test for normal distribution | | | | | | |
| Shapiro-Wilk test | | | | | | |
| W | 0.9170 | 0.8018 | 0.7973 | 0.9496 | 0.8841 | 0.8275 |
| P value | 0.4839 | 0.0610 | 0.0556 | 0.7374 | 0.2882 | 0.1023 |
| Passed normality test (alpha=0.05)' | Yes | Yes | Yes | Yes | Yes | Yes |
| P value summary | ns | ns | ns | ns | ns | ns |

### Figure 1G

| Normality and Lognormality Tests<br>Tabular results | A | B | C | D | E | F |
|---|---|---|---|---|---|---|
| | sham | 6h | 12h | 1d | 3d | 7d |
| Test for normal distribution | | | | | | |
| Shapiro-Wilk test | | | | | | |
| W | 0.9656 | 0.9114 | 0.9446 | 0.7858 | 0.9681 | 0.9291 |
| P value | 0.8619 | 0.4455 | 0.6968 | 0.0436 | 0.8797 | 0.5732 |
| Passed normality test (alpha=0.05)' | Yes | Yes | Yes | No | Yes | Yes |
| P value summary | ns | ns | ns | * | ns | ns |

### Figure 2A

| Normality and Lognormality Tests<br>Tabular results | A | B | C |
|---|---|---|---|
| | Vehicle | Cl-amidine | DNase I |
| Test for normal distribution | | | |
| Shapiro-Wilk test | | | |
| W | 0.8538 | 0.8282 | 0.9829 |
| P value | 0.1690 | 0.1038 | 0.9648 |
| Passed normality test (alpha=0.05)' | Yes | Yes | Yes |
| P value summary | ns | ns | ns |

### Figure 2B

| Normality and Lognormality Tests<br>Tabular results | A | B | C |
|---|---|---|---|
| | Vehicle | Cl-amidine | DNase I |
| Test for normal distribution | | | |
| Shapiro-Wilk test | | | |
| W | 0.8965 | 0.8377 | 0.9603 |
| P value | 0.3536 | 0.1249 | 0.8218 |
| Passed normality test (alpha=0.05)' | Yes | Yes | Yes |
| P value summary | ns | ns | ns |

### Figure 2C

| Normality and Lognormality Tests<br>Tabular results | A | B | C |
|---|---|---|---|
| | Vehicle | Cl-amidine | DNase I |
| Test for normal distribution | | | |
| Shapiro-Wilk test | | | |
| W | 0.9193 | 0.9224 | 0.8502 |
| P value | 0.5001 | 0.5231 | 0.1579 |
| Passed normality test (alpha=0.05)' | Yes | Yes | Yes |
| P value summary | ns | ns | ns |

### Figure 2E (CitH3)

| Normality and Lognormality Tests<br>Tabular results | A | B | C | D |
|---|---|---|---|---|
| | Sham | Vehicle | Cl-amidine | DNase I |
| Test for normal distribution | | | | |
| Shapiro-Wilk test | | | | |
| W | 0.9560 | 0.9360 | 0.8409 | 0.9404 |
| P value | 0.7884 | 0.6274 | 0.1326 | 0.6624 |
| Passed normality test (alpha=0.05)' | Yes | Yes | Yes | Yes |
| P value summary | ns | ns | ns | ns |

### Figure 2E (MPO)

| Normality and Lognormality Tests<br>Tabular results | A | B | C | D |
|---|---|---|---|---|
| | Sham | Vehicle | Cl-amidine | DNase I |
| Test for normal distribution | | | | |
| Shapiro-Wilk test | | | | |
| W | 0.9118 | 0.9713 | 0.9057 | 0.9937 |
| P value | 0.4485 | 0.9007 | 0.4090 | 0.9962 |
| Passed normality test (alpha=0.05)' | Yes | Yes | Yes | Yes |
| P value summary | ns | ns | ns | ns |

### Figure 3B

### Figure 3C

| Normality and Lognormality Tests<br>Tabular results | A | B | C | D |
|---|---|---|---|---|
| | Sham | Vehicle | Cl-amidine | DNase I |
| Test for normal distribution | | | | |
| Shapiro-Wilk test | | | | |
| W | 0.8696 | 0.8987 | 0.9255 | 0.8731 |
| P value | 0.2248 | 0.3665 | 0.5462 | 0.2386 |
| Passed normality test (alpha=0.05)? | Yes | Yes | Yes | Yes |
| P value summary | ns | ns | ns | ns |

| Normality and Lognormality Tests<br>Tabular results | A | B | C |
|---|---|---|---|
| | Vehicle | Cl-amidine | DNase I |
| Test for normal distribution | | | |
| Shapiro-Wilk test | | | |
| W | 0.9366 | 0.9468 | 0.8814 |
| P value | 0.6321 | 0.7142 | 0.2756 |
| Passed normality test (alpha=0.05)? | Yes | Yes | Yes |
| P value summary | ns | ns | ns |

Figure 3D

| Normality and Lognormality Tests<br>Tabular results | A | B | C |
|---|---|---|---|
| | Vehicle | Cl-amidine | DNase I |
| Test for normal distribution | | | |
| Shapiro-Wilk test | | | |
| W | 0.9418 | 0.9328 | 0.9674 |
| P value | 0.6736 | 0.6016 | 0.8742 |
| Passed normality test (alpha=0.05)? | Yes | Yes | Yes |
| P value summary | ns | ns | ns |

Figure 3E

| Normality and Lognormality Tests<br>Tabular results | A | B | C |
|---|---|---|---|
| | Vehicle | Cl-amidine | DNase I |
| Test for normal distribution | | | |
| Shapiro-Wilk test | | | |
| W | 0.9060 | 0.8771 | 0.9459 |
| P value | 0.4105 | 0.2560 | 0.7070 |
| Passed normality test (alpha=0.05)? | Yes | Yes | Yes |
| P value summary | ns | ns | ns |

Figure 3G

| Normality and Lognormality Tests<br>Tabular results | A | B | C | D |
|---|---|---|---|---|
| | Sham | Vehicle | Cl-amidine | DNase I |
| Test for normal distribution | | | | |
| Shapiro-Wilk test | | | | |
| W | 0.9453 | 0.9174 | 0.8763 | 0.8830 |
| P value | 0.7021 | 0.4867 | 0.2525 | 0.2830 |
| Passed normality test (alpha=0.05)? | Yes | Yes | Yes | Yes |
| P value summary | ns | ns | ns | ns |

Figure 3H

| Normality and Lognormality Tests<br>Tabular results | A | B | C | D |
|---|---|---|---|---|
| | Sham | Vehicle | Cl-amidine | DNase I |
| Test for normal distribution | | | | |
| Shapiro-Wilk test | | | | |
| W | 0.6412 | 0.9581 | 0.9173 | 0.8599 |
| P value | 0.0005 | 0.7922 | 0.4082 | 0.1199 |
| Passed normality test (alpha=0.05)? | No | Yes | Yes | Yes |
| P value summary | *** | ns | ns | ns |

Figure 3I

| Normality and Lognormality Tests<br>Tabular results | A | B | C | D |
|---|---|---|---|---|
| | Sham | Vehicle | Cl-amidine | DNase I |
| Test for normal distribution | | | | |
| Shapiro-Wilk test | | | | |
| W | 0.6412 | 0.9116 | 0.8599 | 0.9116 |
| P value | 0.0005 | 0.3657 | 0.1199 | 0.3657 |
| Passed normality test (alpha=0.05)? | No | Yes | Yes | Yes |
| P value summary | *** | ns | ns | ns |

Figure 3J

| Normality and Lognormality Tests<br>Tabular results | A | B | C | D |
|---|---|---|---|---|
| | Sham | Vehicle | Cl-amidine | DNase I |
| Test for normal distribution | | | | |
| Shapiro-Wilk test | | | | |
| W | 0.5659 | 0.6412 | 0.8104 | 0.8715 |
| P value | <0.0001 | 0.0005 | 0.0370 | 0.1560 |
| Passed normality test (alpha=0.05)? | No | No | No | Yes |
| P value summary | **** | *** | * | ns |

Figure 4A

| Normality and Lognormality Tests<br>Tabular results | A | B | C | D | E | F |
|---|---|---|---|---|---|---|
| | Sham | SBI 6h | SBI 12h | SBI 1d | SBI 3d | SBI 7d |
| Test for normal distribution | | | | | | |
| Shapiro-Wilk test | | | | | | |
| W | 0.9207 | 0.8276 | 0.8840 | 0.8723 | 0.9190 | 0.8646 |
| P value | 0.5105 | 0.1026 | 0.2881 | 0.2356 | 0.4980 | 0.2055 |
| Passed normality test (alpha=0.05)? | Yes | Yes | Yes | Yes | Yes | Yes |
| P value summary | ns | ns | ns | ns | ns | ns |

Figure 4B

| Normality and Lognormality Tests<br>Tabular results | A | B |
|---|---|---|
| | CitH3 | IFN-β |
| Test for normal distribution | | |
| Shapiro-Wilk test | | |
| W | 0.8052 | 0.8723 |
| P value | 0.0654 | 0.2356 |
| Passed normality test (alpha=0.05)? | Yes | Yes |
| P value summary | ns | ns |

Figure 4C

| Normality and Lognormality Tests<br>Tabular results | A | B |
|---|---|---|
| | MPO-DNA | IFN-β |
| Test for normal distribution | | |
| Shapiro-Wilk test | | |
| W | 0.9496 | 0.8723 |
| P value | 0.7374 | 0.2356 |
| Passed normality test (alpha=0.05)? | Yes | Yes |
| P value summary | ns | ns |

Figure 4E

| Normality and Lognormality Tests<br>Tabular results | A | B | C | D | E | F |
|---|---|---|---|---|---|---|
| | Sham | SBI 6h | SBI 12h | SBI 1d | SBI 3d | SBI 7d |
| Test for normal distribution | | | | | | |
| Shapiro-Wilk test | | | | | | |
| W | 0.8423 | 0.9659 | 0.9141 | 0.9906 | 0.9499 | 0.9717 |
| P value | 0.1363 | 0.8637 | 0.4636 | 0.9906 | 0.7396 | 0.9037 |
| Passed normality test (alpha=0.05)? | Yes | Yes | Yes | Yes | Yes | Yes |
| P value summary | ns | ns | ns | ns | ns | ns |

Figure 5A

| Normality and Lognormality Tests<br>Tabular results | A | B |
|---|---|---|
| | Vehicle | RU.521 |
| Test for normal distribution | | |
| Shapiro-Wilk test | | |
| W | 0.8989 | 0.9841 |
| P value | 0.3675 | 0.9698 |
| Passed normality test (alpha=0.05)? | Yes | Yes |
| P value summary | ns | ns |

Figure 5B

| Normality and Lognormality Tests<br>Tabular results | A | B |
|---|---|---|
| | Vehicle | RU.521 |
| Test for normal distribution | | |
| Shapiro-Wilk test | | |
| W | 0.8814 | 0.9364 |
| P value | 0.2755 | 0.6300 |
| Passed normality test (alpha=0.05)? | Yes | Yes |
| P value summary | ns | ns |

## Figure 5C

| Normality and Lognormality Tests<br>Tabular results | A<br>Vehicle | B<br>RU.521 |
|---|---|---|
| Test for normal distribution | | |
| Shapiro-Wilk test | | |
| W | 0.9859 | 0.9427 |
| P value | 0.9767 | 0.6812 |
| Passed normality test (alpha=0.05)' | Yes | Yes |
| P value summary | ns | ns |

## Figure 5D

| Normality and Lognormality Tests<br>Tabular results | A<br>Vehicle | B<br>RU.521 |
|---|---|---|
| Test for normal distribution | | |
| Shapiro-Wilk test | | |
| W | 0.9135 | 0.9853 |
| P value | 0.4596 | 0.9748 |
| Passed normality test (alpha=0.05)' | Yes | Yes |
| P value summary | ns | ns |

## Figure 5F

| Normality and Lognormality Tests<br>Tabular results | A<br>Vehicle | B<br>RU.521 |
|---|---|---|
| Test for normal distribution | | |
| Shapiro-Wilk test | | |
| W | 0.9484 | 0.9470 |
| P value | 0.7272 | 0.7163 |
| Passed normality test (alpha=0.05)' | Yes | Yes |
| P value summary | ns | ns |

## Figure 5G

| Normality and Lognormality Tests<br>Tabular results | A<br>Vehicle | B<br>RU.521 |
|---|---|---|
| Test for normal distribution | | |
| Shapiro-Wilk test | | |
| W | 0.8272 | 0.8104 |
| P value | 0.0555 | 0.0370 |
| Passed normality test (alpha=0.05)' | Yes | No |
| P value summary | ns | * |

## Figure 5H

| Normality and Lognormality Tests<br>Tabular results | A<br>Vehicle | B<br>RU.521 |
|---|---|---|
| Test for normal distribution | | |
| Shapiro-Wilk test | | |
| W | 0.8104 | 0.6930 |
| P value | 0.0370 | 0.0019 |
| Passed normality test (alpha=0.05)' | No | No |
| P value summary | * | ** |

## Figure 5I

| Normality and Lognormality Tests<br>Tabular results | A<br>Vehicle | B<br>RU.521 |
|---|---|---|
| Test for normal distribution | | |
| Shapiro-Wilk test | | |
| W | 0.6013 | 0.8352 |
| P value | 0.0002 | 0.0672 |
| Passed normality test (alpha=0.05)' | No | Yes |
| P value summary | *** | ns |

## Figure 6A

| Normality and Lognormality Tests<br>Tabular results | A<br>Vehicle | B<br>DNase I |
|---|---|---|
| Test for normal distribution | | |
| Shapiro-Wilk test | | |
| W | 0.9141 | 0.9356 |
| P value | 0.4638 | 0.6244 |
| Passed normality test (alpha=0.05)' | Yes | Yes |
| P value summary | ns | ns |

## Figure 6B

| Normality and Lognormality Tests<br>Tabular results | A<br>Vehicle | B<br>RU.521 |
|---|---|---|
| Test for normal distribution | | |
| Shapiro-Wilk test | | |
| W | 0.8409 | 0.9723 |
| P value | 0.1325 | 0.9078 |
| Passed normality test (alpha=0.05)' | Yes | Yes |
| P value summary | ns | ns |

## Figure 6C

| Normality and Lognormality Tests<br>Tabular results | A<br>Vehicle | B<br>RU.521 |
|---|---|---|
| Test for normal distribution | | |
| Shapiro-Wilk test | | |
| W | 0.8913 | 0.9168 |
| P value | 0.3248 | 0.4828 |
| Passed normality test (alpha=0.05)' | Yes | Yes |
| P value summary | ns | ns |

## Figure 6E

| Normality and Lognormality Tests<br>Tabular results | A<br>Sham | B<br>Vehicle | C<br>DNase I | D<br>DNase I+cGAMP |
|---|---|---|---|---|
| Test for normal distribution | | | | |
| Shapiro-Wilk test | | | | |
| W | 0.8587 | 0.9299 | 0.9675 | 0.8759 |
| P value | 0.1849 | 0.5797 | 0.8753 | 0.2509 |
| Passed normality test (alpha=0.05)' | Yes | Yes | Yes | Yes |
| P value summary | ns | ns | ns | ns |

## Figure 6F

| Normality and Lognormality Tests<br>Tabular results | A<br>Sham | B<br>Vehicle | C<br>DNase I | D<br>DNase I+cGAMP |
|---|---|---|---|---|
| Test for normal distribution | | | | |
| Shapiro-Wilk test | | | | |
| W | 0.8653 | 0.8675 | 0.8715 | 0.9458 |
| P value | 0.2080 | 0.2165 | 0.2324 | 0.7059 |
| Passed normality test (alpha=0.05)' | Yes | Yes | Yes | Yes |
| P value summary | ns | ns | ns | ns |

## Figure 6H

| Normality and Lognormality Tests<br>Tabular results | A<br>DNase I+Vehicle | B<br>DNase I+cGAMP |
|---|---|---|
| Test for normal distribution | | |
| Shapiro-Wilk test | | |
| W | 0.9696 | 0.8487 |
| P value | 0.8897 | 0.1538 |
| Passed normality test (alpha=0.05)' | Yes | Yes |
| P value summary | ns | ns |

## Figure 6I (IL-6)

| Normality and Lognormality Tests<br>Tabular results | A<br>DNase I+Vehicle | B<br>DNase I+cGAMP |
|---|---|---|
| Test for normal distribution | | |
| Shapiro-Wilk test | | |
| W | 0.8158 | 0.8919 |
| P value | 0.0812 | 0.3281 |
| Passed normality test (alpha=0.05)' | Yes | Yes |
| P value summary | ns | ns |

## Figure 6I (TNF)

| Normality and Lognormality Tests<br>Tabular results | A<br>DNase I+Vehicle | B<br>DNase I+cGAMP |
|---|---|---|
| Test for normal distribution | | |
| Shapiro-Wilk test | | |
| W | 0.9168 | 0.8882 |
| P value | 0.4829 | 0.3090 |
| Passed normality test (alpha=0.05)' | Yes | Yes |
| P value summary | ns | ns |

Figure 6K

| Normality and Lognormality Tests<br>Tabular results | A<br>DNase I+Vehicle | B<br>DNase I+cGAMP |
|---|---|---|
| Test for normal distribution | | |
| Shapiro-Wilk test | | |
| W | 0.9359 | 0.7974 |
| P value | 0.6261 | 0.0556 |
| Passed normality test (alpha=0.05)' | Yes | Yes |
| P value summary | ns | ns |

Figure 6L

| Normality and Lognormality Tests<br>Tabular results | A<br>DNase I+Vehicle | B<br>DNase I+cGAMP |
|---|---|---|
| Test for normal distribution | | |
| Shapiro-Wilk test | | |
| W | 0.8570 | 0.8219 |
| P value | 0.1790 | 0.0917 |
| Passed normality test (alpha=0.05)' | Yes | Yes |
| P value summary | ns | ns |

Figure 6M (1d)

| Normality and Lognormality Tests<br>Tabular results | A<br>DNase I+Vehicle | B<br>DNase I+cGAMP |
|---|---|---|
| Test for normal distribution | | |
| Shapiro-Wilk test | | |
| W | 0.6412 | 0.8104 |
| P value | 0.0005 | 0.0370 |
| Passed normality test (alpha=0.05)' | No | No |
| P value summary | *** | * |

Figure 6M (3d)

| Normality and Lognormality Tests<br>Tabular results | A<br>DNase I+Vehicle | B<br>DNase I+cGAMP |
|---|---|---|
| Test for normal distribution | | |
| Shapiro-Wilk test | | |
| W | 0.8148 | 0.7238 |
| P value | 0.0411 | 0.0042 |
| Passed normality test (alpha=0.05)' | No | No |
| P value summary | * | ** |

Figure 6M (7d)

| Normality and Lognormality Tests<br>Tabular results | A<br>DNase I+Vehicle | B<br>DNase I+cGAMP |
|---|---|---|
| Test for normal distribution | | |
| Shapiro-Wilk test | | |
| W | 0.7823 | 0.8973 |
| P value | 0.0185 | 0.2730 |
| Passed normality test (alpha=0.05)' | No | Yes |
| P value summary | * | ns |

Figure 7C (IL-6)

| Normality and Lognormality Tests<br>Tabular results | A<br>Sham | B<br>Vehicle | C<br>DNase I |
|---|---|---|---|
| Test for normal distribution | | | |
| Shapiro-Wilk test | | | |
| W | 0.9215 | 0.8249 | 0.9388 |
| P value | 0.5165 | 0.0973 | 0.6498 |
| Passed normality test (alpha=0.05)' | Yes | Yes | Yes |
| P value summary | ns | ns | ns |

Figure 7C (TNF)

| Normality and Lognormality Tests<br>Tabular results | A<br>Sham | B<br>Vehicle | C<br>DNase I |
|---|---|---|---|
| Test for normal distribution | | | |
| Shapiro-Wilk test | | | |
| W | 0.9250 | 0.9576 | 0.9143 |
| P value | 0.5417 | 0.8010 | 0.4655 |
| Passed normality test (alpha=0.05)' | Yes | Yes | Yes |
| P value summary | ns | ns | ns |

Figure 7E (IL-6)

| Normality and Lognormality Tests<br>Tabular results | A<br>DNase I +Vehicle | B<br>DNase I+cGAMP |
|---|---|---|
| Test for normal distribution | | |
| Shapiro-Wilk test | | |
| W | 0.9731 | 0.9555 |
| P value | 0.9126 | 0.7846 |
| Passed normality test (alpha=0.05)' | Yes | Yes |
| P value summary | ns | ns |

Figure 7E (TNF)

| Normality and Lognormality Tests<br>Tabular results | A<br>DNase I +Vehicle | B<br>DNase I+cGAMP |
|---|---|---|
| Test for normal distribution | | |
| Shapiro-Wilk test | | |
| W | 0.9088 | 0.9717 |
| P value | 0.4287 | 0.9035 |
| Passed normality test (alpha=0.05)' | Yes | Yes |
| P value summary | ns | ns |

Figure 8A

| Normality and Lognormality Tests<br>Tabular results | A<br>Sham | B<br>SBI |
|---|---|---|
| Test for normal distribution | | |
| Shapiro-Wilk test | | |
| W | 0.8789 | 0.9792 |
| P value | 0.2639 | 0.9476 |
| Passed normality test (alpha=0.05)' | Yes | Yes |
| P value summary | ns | ns |

Figure 8B

| Normality and Lognormality Tests<br>Tabular results | A<br>sham | B<br>Vehicle | C<br>Vitamin C 100 | D<br>Vitamin C 200 | E<br>Vitamin C 500 |
|---|---|---|---|---|---|
| Test for normal distribution | | | | | |
| Shapiro-Wilk test | | | | | |
| W | 0.9030 | 0.9376 | 0.8855 | 0.9302 | 0.9498 |
| P value | 0.3919 | 0.6399 | 0.2953 | 0.5815 | 0.7388 |
| Passed normality test (alpha=0.05)' | Yes | Yes | Yes | Yes | Yes |
| P value summary | ns | ns | ns | ns | ns |

Figure 8C

Figure 8D

| Normality and Lognormality Tests<br>Tabular results | A<br>Vehicle | B<br>Vitamin C 100 | C<br>Vitamin C 200 | D<br>Vitamin C 500 |
|---|---|---|---|---|
| Test for normal distribution | | | | |
| Shapiro-Wilk test | | | | |
| W | 0.9564 | 0.8881 | 0.8423 | 0.9623 |
| P value | 0.7912 | 0.3084 | 0.1361 | 0.8370 |
| Passed normality test (alpha=0.05)' | Yes | Yes | Yes | Yes |
| P value summary | ns | ns | ns | ns |

| Normality and Lognormality Tests<br>Tabular results | A<br>Vehicle | B<br>Vitamin C 100 | C<br>Vitamin C 200 | D<br>Vitamin C 500 |
|---|---|---|---|---|
| Test for normal distribution | | | | |
| Shapiro-Wilk test | | | | |
| W | 0.8636 | 0.9618 | 0.9382 | 0.9639 |
| P value | 0.2020 | 0.8332 | 0.6449 | 0.8496 |
| Passed normality test (alpha=0.05)' | Yes | Yes | Yes | Yes |
| P value summary | ns | ns | ns | ns |

Figure 8F

| Normality and Lognormality Tests<br>Tabular results | A<br>Vehicle | B<br>Vitamin C 100 | C<br>Vitamin C 200 | D<br>Vitamin C 500 |
|---|---|---|---|---|
| Test for normal distribution | | | | |
| Shapiro-Wilk test | | | | |
| W | 0.9615 | 0.9236 | 0.9879 | 0.8424 |
| P value | 0.8314 | 0.5315 | 0.9835 | 0.1366 |
| Passed normality test (alpha=0.05)' | Yes | Yes | Yes | Yes |
| P value summary | ns | ns | ns | ns |

**Homogeneity of variance assessment (Except for the red-marked data, all conform to the homogeneity of variance)**

Figure 1A

| Brown-Forsythe test | |
|---|---|
| F (DFn, DFd) | 1.757 (5, 30) |
| P value | 0.1520 |
| P value summary | ns |
| Are SDs significantly different (P < 0.05)? | No |

Figure 1B

| Brown-Forsythe test | |
|---|---|
| F (DFn, DFd) | 0.9052 (5, 30) |
| P value | 0.4907 |
| P value summary | ns |
| Are SDs significantly different (P < 0.05)? | No |

Figure 1C

| Brown-Forsythe test | |
|---|---|
| F (DFn, DFd) | 3.748 (5, 30) |
| P value | 0.0094 |
| P value summary | ** |
| Are SDs significantly different (P < 0.05)? | Yes |

Figure 1D

| Brown-Forsythe test | |
|---|---|
| F (DFn, DFd) | 1.313 (5, 30) |
| P value | 0.2850 |
| P value summary | ns |
| Are SDs significantly different (P < 0.05)? | No |

Figure 1E

| Brown-Forsythe test | |
|---|---|
| F (DFn, DFd) | 6.867 (5, 30) |
| P value | 0.0002 |
| P value summary | *** |
| Are SDs significantly different (P < 0.05)? | Yes |

Figure 1G

| Brown-Forsythe test | |
|---|---|
| F (DFn, DFd) | 0.5301 (5, 30) |
| P value | 0.7517 |
| P value summary | ns |
| Are SDs significantly different (P < 0.05)? | No |

Figure 2A

| Brown-Forsythe test | |
|---|---|
| F (DFn, DFd) | 0.3281 (2, 15) |
| P value | 0.7253 |
| P value summary | ns |
| Are SDs significantly different (P < 0.05)? | No |

Figure 2B

| Brown-Forsythe test | |
|---|---|
| F (DFn, DFd) | 34.62 (2, 15) |
| P value | <0.0001 |
| P value summary | **** |
| Are SDs significantly different (P < 0.05)? | Yes |

Figure 2C

| Brown-Forsythe test | |
|---|---|
| F (DFn, DFd) | 0.1493 (2, 15) |
| P value | 0.8626 |
| P value summary | ns |
| Are SDs significantly different (P < 0.05)? | No |

Figure 2E (CitH3)

| Brown-Forsythe test | |
|---|---|
| F (DFn, DFd) | 4.543 (3, 20) |
| P value | 0.0139 |
| P value summary | * |
| Are SDs significantly different (P < 0.05)? | Yes |

Figure 2E (MPO)

| Brown-Forsythe test | |
|---|---|
| F (DFn, DFd) | 1.815 (3, 20) |
| P value | 0.1769 |
| P value summary | ns |
| Are SDs significantly different (P < 0.05)? | No |

Figure 3B

| Brown-Forsythe test | |
|---|---|
| F (DFn, DFd) | 2.113 (3, 20) |
| P value | 0.1306 |
| P value summary | ns |
| Are SDs significantly different (P < 0.05)' | No |

Figure 3C

| Brown-Forsythe test | |
|---|---|
| F (DFn, DFd) | 2.351 (2, 15) |
| P value | 0.1294 |
| P value summary | ns |
| Are SDs significantly different (P < 0.05)' | No |

Figure 3D

| Brown-Forsythe test | |
|---|---|
| F (DFn, DFd) | 0.8231 (2, 15) |
| P value | 0.4580 |
| P value summary | ns |
| Are SDs significantly different (P < 0.05)' | No |

Figure 3E

| Brown-Forsythe test | |
|---|---|
| F (DFn, DFd) | 0.8476 (2, 15) |
| P value | 0.4480 |
| P value summary | ns |
| Are SDs significantly different (P < 0.05)' | No |

Figure 3G

| Brown-Forsythe test | |
|---|---|
| F (DFn, DFd) | 0.7641 (3, 20) |
| P value | 0.5275 |
| P value summary | ns |
| Are SDs significantly different (P < 0.05)' | No |

Figure 3H

| Brown-Forsythe test | |
|---|---|
| F (DFn, DFd) | 0.7636 (3, 28) |
| P value | 0.5240 |
| P value summary | ns |
| Are SDs significantly different (P < 0.05)' | No |

Figure 3I

| Brown-Forsythe test | |
|---|---|
| F (DFn, DFd) | 1.127 (3, 28) |
| P value | 0.3549 |
| P value summary | ns |
| Are SDs significantly different (P < 0.05)' | No |

Figure 3J

| Brown-Forsythe test | |
|---|---|
| F (DFn, DFd) | 0.6073 (3, 28) |
| P value | 0.6158 |
| P value summary | ns |
| Are SDs significantly different (P < 0.05)' | No |

Figure 4A

| Brown-Forsythe test | |
|---|---|
| F (DFn, DFd) | 1.302 (5, 30) |
| P value | 0.2895 |
| P value summary | ns |
| Are SDs significantly different (P < 0.05)' | No |

Figure 4B

| F test to compare variances | |
|---|---|
| F, DFn, Dfd | 1.623, 5, 5 |
| P value | 0.6079 |
| P value summary | ns |
| Significantly different (P < 0.05)? | No |

Figure 4C

| F test to compare variances | |
|---|---|
| F, DFn, Dfd | 89.72, 5, 5 |
| P value | 0.0001 |
| P value summary | *** |
| Significantly different (P < 0.05)? | Yes |

Figure 4E

| Brown-Forsythe test | |
|---|---|
| F (DFn, DFd) | 1.970 (5, 30) |
| P value | 0.1120 |
| P value summary | ns |
| Are SDs significantly different (P < 0.05)' | No |

Figure 5A

| F test to compare variances | |
|---|---|
| F, DFn, Dfd | 5.329, 5, 5 |
| P value | 0.0901 |
| P value summary | ns |
| Significantly different (P < 0.05)? | No |

Figure 5B

| F test to compare variances | |
|---|---|
| F, DFn, Dfd | 1.738, 5, 5 |
| P value | 0.5590 |
| P value summary | ns |
| Significantly different (P < 0.05)? | No |

Figure 5C

Figure 5D

| F test to compare variances | |
|---|---|
| F, DFn, Dfd | 1.334, 5, 5 |
| P value | 0.7594 |
| P value summary | ns |
| Significantly different (P < 0.05)? | No |

| F test to compare variances | |
|---|---|
| F, DFn, Dfd | 1.375, 5, 5 |
| P value | 0.7350 |
| P value summary | ns |
| Significantly different (P < 0.05)? | No |

Figure 5F

Figure 5G

| F test to compare variances | |
|---|---|
| F, DFn, Dfd | 3.416, 5, 5 |
| P value | 0.2038 |
| P value summary | ns |
| Significantly different (P < 0.05)? | No |

| F test to compare variances | |
|---|---|
| F, DFn, Dfd | 1.217, 7, 7 |
| P value | 0.8019 |
| P value summary | ns |
| Significantly different (P < 0.05)? | No |

Figure 5H

Figure 5I

| F test to compare variances | |
|---|---|
| F, DFn, Dfd | 2.043, 7, 7 |
| P value | 0.3664 |
| P value summary | ns |
| Significantly different (P < 0.05)? | No |

| F test to compare variances | |
|---|---|
| F, DFn, Dfd | 1.258, 7, 7 |
| P value | 0.7697 |
| P value summary | ns |
| Significantly different (P < 0.05)? | No |

Figure 6A

Figure 6B

| F test to compare variances | |
|---|---|
| F, DFn, Dfd | 1.263, 5, 5 |
| P value | 0.8043 |
| P value summary | ns |
| Significantly different (P < 0.05)? | No |

| F test to compare variances | |
|---|---|
| F, DFn, Dfd | 4.554, 5, 5 |
| P value | 0.1216 |
| P value summary | ns |
| Significantly different (P < 0.05)? | No |

Figure 6C

Figure 6E

| F test to compare variances | |
|---|---|
| F, DFn, Dfd | 1.650, 5, 5 |
| P value | 0.5962 |
| P value summary | ns |
| Significantly different (P < 0.05)? | No |

| Brown-Forsythe test | |
|---|---|
| F (DFn, DFd) | 2.187 (3, 20) |
| P value | 0.1213 |
| P value summary | ns |
| Are SDs significantly different (P < 0.05)? | No |

Figure 6F

Figure 6H

| Brown-Forsythe test | |
|---|---|
| F (DFn, DFd) | 0.2012 (3, 20) |
| P value | 0.8943 |
| P value summary | ns |
| Are SDs significantly different (P < 0.05)? | No |

| F test to compare variances | |
|---|---|
| F, DFn, Dfd | 1.475, 5, 5 |
| P value | 0.6801 |
| P value summary | ns |
| Significantly different (P < 0.05)? | No |

Figure 6I (IL-6)

Figure 6I (TNF)

| F test to compare variances | |
|---|---|
| F, DFn, Dfd | 1.044, 5, 5 |
| P value | 0.9638 |
| P value summary | ns |
| Significantly different (P < 0.05)? | No |

| F test to compare variances | |
|---|---|
| F, DFn, Dfd | 1.144, 5, 5 |
| P value | 0.8866 |
| P value summary | ns |
| Significantly different (P < 0.05)? | No |

Figure 6K

Figure 6L

| F test to compare variances | |
|---|---|
| F, DFn, Dfd | 1.211, 5, 5 |
| P value | 0.8391 |
| P value summary | ns |
| Significantly different (P < 0.05)? | No |

| F test to compare variances | |
|---|---|
| F, DFn, Dfd | 1.327, 5, 5 |
| P value | 0.7636 |
| P value summary | ns |
| Significantly different (P < 0.05)? | No |

Figure 6M (1d)

Figure 6M (3d)

| F test to compare variances | |
|---|---|
| F, DFn, Dfd | 1.533, 7, 7 |
| P value | 0.5866 |
| P value summary | ns |
| Significantly different (P < 0.05)? | No |

| F test to compare variances | |
|---|---|
| F, DFn, Dfd | 2.500, 7, 7 |
| P value | 0.2498 |
| P value summary | ns |
| Significantly different (P < 0.05)? | No |

Figure 6M (7d)

| F test to compare variances | |
|---|---|
| F, DFn, Dfd | 2.545, 7, 7 |
| P value | 0.2409 |
| P value summary | ns |
| Significantly different (P < 0.05)? | No |

Figure 7C (IL-6)

| Brown-Forsythe test | |
|---|---|
| F (DFn, DFd) | 0.5210 (2, 15) |
| P value | 0.6043 |
| P value summary | ns |
| Are SDs significantly different (P < 0.05)? | No |

Figure 7C (TNF)

| Brown-Forsythe test | |
|---|---|
| F (DFn, DFd) | 3.321 (2, 15) |
| P value | 0.0640 |
| P value summary | ns |
| Are SDs significantly different (P < 0.05)? | No |

Figure 7E (IL-6)

| F test to compare variances | |
|---|---|
| F, DFn, Dfd | 2.389, 5, 5 |
| P value | 0.3612 |
| P value summary | ns |
| Significantly different (P < 0.05)? | No |

Figure 7E (TNF)

| F test to compare variances | |
|---|---|
| F, DFn, Dfd | 2.759, 5, 5 |
| P value | 0.2896 |
| P value summary | ns |
| Significantly different (P < 0.05)? | No |

Figure 8A

| F test to compare variances | |
|---|---|
| F, DFn, Dfd | 1.468, 5, 5 |
| P value | 0.6840 |
| P value summary | ns |
| Significantly different (P < 0.05)? | No |

Figure 8B

| Brown-Forsythe test | |
|---|---|
| F (DFn, DFd) | 0.7448 (4, 25) |
| P value | 0.5706 |
| P value summary | ns |
| Are SDs significantly different (P < 0.05)? | No |

Figure 8C

| Brown-Forsythe test | |
|---|---|
| F (DFn, DFd) | 1.195 (3, 20) |
| P value | 0.3369 |
| P value summary | ns |
| Are SDs significantly different (P < 0.05)? | No |

Figure 8D

| Brown-Forsythe test | |
|---|---|
| F (DFn, DFd) | 0.1945 (3, 20) |
| P value | 0.8989 |
| P value summary | ns |
| Are SDs significantly different (P < 0.05)? | No |

Figure 8F

| Brown-Forsythe test | |
|---|---|
| F (DFn, DFd) | 2.117 (3, 20) |
| P value | 0.1301 |
| P value summary | ns |
| Are SDs significantly different (P < 0.05)? | No |

**Results of statistical tests**

Figure 1A

| | Z | P value |
|---|---|---|
| Sham vs. SBI 6h | 0.3836 | 0.7013 |
| Sham vs. SBI 12h | 1.671 | 0.0946 |
| Sham vs. SBI 1d | 3.644 | 0.0003 |
| Sham vs. SBI 3d | 4.631 | <0.0001 |
| Sham vs. SBI 7d | 2.658 | 0.0079 |

Figure 1B

| | Z | P value |
|---|---|---|
| Sham vs. SBI 6h | 0.7125 | 0.4762 |
| Sham vs. SBI 12h | 2.274 | 0.0229 |
| Sham vs. SBI 1d | 3.809 | 0.0001 |
| Sham vs. SBI 3d | 4.796 | <0.0001 |
| Sham vs. SBI 7d | 2.384 | 0.0171 |

Figure 1C

| | Z | P value |
|---|---|---|
| Sham vs. SBI 6h | 0.2192 | 0.8265 |
| Sham vs. SBI 12h | 0.8768 | 0.3806 |
| Sham vs. SBI 1d | 2.384 | 0.0171 |
| Sham vs. SBI 3d | 4.000 | <0.0001 |
| Sham vs. SBI 7d | 1.726 | 0.0843 |

Figure 1D

| | Z | P value |
|---|---|---|
| Sham vs. SBI 6h | 0.7672 | 0.4430 |
| Sham vs. SBI 12h | 2.576 | 0.0100 |
| Sham vs. SBI 1d | 4.795 | <0.0001 |
| Sham vs. SBI 3d | 3.809 | 0.0001 |
| Sham vs. SBI 7d | 2.028 | 0.0426 |

Figure 1E

| | Z | P value |
|---|---|---|
| Sham vs. SBI 6h | 0.6851 | 0.4933 |
| Sham vs. SBI 12h | 2.480 | 0.0131 |
| Sham vs. SBI 1d | 4.631 | <0.0001 |
| Sham vs. SBI 3d | 3.864 | 0.0001 |
| Sham vs. SBI 7d | 1.987 | 0.0469 |

Figure 1G

| | Z | P value |
|---|---|---|
| Sham vs. SBI 6h | 0.7124 | 0.4762 |
| Sham vs. SBI 12h | 0.8494 | 0.3957 |
| Sham vs. SBI 1d | 1.589 | 0.1120 |
| Sham vs. SBI 3d | 4.110 | <0.0001 |
| Sham vs. SBI 7d | 3.096 | 0.0020 |

Figure 2A

| | Z | P value |
|---|---|---|
| Vehicle vs. Cl-amidine | 2.812 | 0.0049 |
| Vehicle vs. DNase I | 3.028 | 0.0025 |

Figure 2B

| | Z | P value |
|---|---|---|
| Vehicle vs. Cl-amidine | 2.433 | 0.0150 |
| Vehicle vs. DNase I | 3.407 | 0.0007 |

Figure 2C

| | Z | P value |
|---|---|---|
| Vehicle vs. Cl-amidine | 2.758 | 0.0058 |
| Vehicle vs. DNase I | 3.082 | 0.0021 |

Figure 2E (CitH3)

| | Z | P value |
|---|---|---|
| Sham vs. Vehicle | 4.287 | <0.0001 |
| Vehicle vs. Cl-amidine | 2.245 | 0.0247 |
| Vehicle vs. DNase I | 2.327 | 0.0200 |

Figure 2E (MPO)

| | Z | P value |
|---|---|---|
| Sham vs. Vehicle | 4.409 | <0.0001 |
| Vehicle vs. Cl-amidine | 2.123 | 0.0338 |
| Vehicle vs. DNase I | 2.286 | 0.0222 |

Figure 3B

| | Z | P value |
|---|---|---|
| Sham vs. Vehicle | 4.409 | <0.0001 |
| Vehicle vs. Cl-amidine | 2.041 | 0.0412 |
| Vehicle vs. DNase I | 2.368 | 0.0179 |

Figure 3C

| | Z | P value |
|---|---|---|

| | | |
|---|---|---|
| Vehicle vs. Cl-amidine | 2.596 | 0.0094 |
| Vehicle vs. DNase I | 3.244 | 0.0012 |

Figure 3D

| | Z | P value |
|---|---|---|
| Vehicle vs. Cl-amidine | 2.433 | 0.0150 |
| Vehicle vs. DNase I | 3.407 | 0.0007 |

Figure 3E

| | Z | P value |
|---|---|---|
| Vehicle vs. Cl-amidine | 2.758 | 0.0058 |
| Vehicle vs. DNase I | 3.082 | 0.0021 |

Figure 3G

| | Z | P value |
|---|---|---|
| Sham vs. Vehicle | 4.409 | <0.0001 |
| Vehicle vs. Cl-amidine | 2.082 | 0.0373 |
| Vehicle vs. DNase I | 2.327 | 0.0200 |

Figure 3H

| | Z | P value |
|---|---|---|
| Sham vs. Vehicle | 4.093 | <0.0001 |
| Vehicle vs. Cl-amidine | 0.7454 | 0.4560 |
| Vehicle vs. DNase I | 1.125 | 0.2606 |

Figure 3I

| | Z | P value |
|---|---|---|
| Sham vs. Vehicle | 5.139 | <0.0001 |
| Vehicle vs. Cl-amidine | 2.476 | 0.0133 |
| Vehicle vs. DNase I | 2.610 | 0.0091 |

Figure 3J

| | Z | P value |
|---|---|---|
| Sham vs. Vehicle | 5.201 | <0.0001 |
| Vehicle vs. Cl-amidine | 2.546 | 0.0109 |
| Vehicle vs. DNase I | 2.655 | 0.0079 |

Figure 4A

| | Z | P value |
|---|---|---|
| Sham vs. SBI 6h | 0.8220 | 0.4111 |
| Sham vs. SBI 12h | 2.356 | 0.0185 |
| Sham vs. SBI 1d | 4.685 | <0.0001 |
| Sham vs. SBI 3d | 4.028 | <0.0001 |

| | | |
|---|---|---|
| Sham vs. SBI 7d | 2.411 | 0.0159 |

Figure 4B

| | r | P value |
|---|---|---|
| | 0.9725 | 0.0011 |

Figure 4C

| | r | P value |
|---|---|---|
| | 0.9574 | 0.0027 |

Figure 4E

| | Z | P value |
|---|---|---|
| Sham vs. SBI 6h | 0.4932 | 0.6219 |
| Sham vs. SBI 12h | 0.7124 | 0.4762 |
| Sham vs. SBI 1d | 1.891 | 0.0587 |
| Sham vs. SBI 3d | 3.891 | <0.0001 |
| Sham vs. SBI 7d | 2.713 | 0.0067 |

Figure 5A

| | U | P value |
|---|---|---|
| Vehicle vs. RU.521 | 0 | 0.0022 |

Figure 5B

| | U | P value |
|---|---|---|
| Vehicle vs. RU.521 | 0 | 0.0022 |

Figure 5C

| | U | P value |
|---|---|---|
| Vehicle vs. RU.521 | 0 | 0.0022 |

Figure 5D

| | U | P value |
|---|---|---|
| Vehicle vs. RU.521 | 0 | 0.0022 |

Figure 5F

| | U | P value |
|---|---|---|
| Vehicle vs. RU.521 | 0 | 0.0022 |

Figure 5G

| | U | P value |
|---|---|---|
| Vehicle vs. RU.521 | 17.50 | 0.1699 |

Figure 5H

| | U | P value |
|---|---|---|
| Vehicle vs. RU.521 | 0 | 0.0002 |

Figure 5I

| | U | P value |
|---|---|---|
| Vehicle vs. RU.521 | 0 | 0.0002 |

Figure 6A

| | U | P value |
|---|---|---|
| Vehicle vs. DNase I | 0 | 0.0022 |

Figure 6B

| | U | P value |
|---|---|---|
| Vehicle vs. RU.521 | 16 | 0.8182 |

Figure 6C

| | U | P value |
|---|---|---|
| Vehicle vs. RU.521 | 15.50 | 0.7316 |

Figure 6E

| | Z | P value |
|---|---|---|
| Sham vs. Vehicle | 3.715 | 0.0002 |
| Vehicle vs. DNase I | 2.327 | 0.0200 |
| DNase I vs. DNase I+cGAMP | 2.164 | 0.0305 |

Figure 6F

| | Z | P value |
|---|---|---|
| Sham vs. Vehicle | 3.348 | 0.0008 |
| Vehicle vs. DNase I | 3.021 | 0.0025 |
| DNase I vs. DNase I+cGAMP | 2.531 | 0.0114 |

Figure 6H

| | U | P value |
|---|---|---|
| DNase I+Vehicle vs. DNase I+cGAMP | 0 | 0.0022 |

Figure 6I (IL-6)

| | U | P value |
|---|---|---|
| DNase I+Vehicle vs. DNase I+cGAMP | 0 | 0.0020 |

Figure 6I (TNF)

| | U | P value |
|---|---|---|

| | | |
|---|---|---|
| DNase I+Vehicle vs. DNase I+cGAMP | 0 | 0.0022 |

Figure 6K

| | U | P value |
|---|---|---|
| DNase I+Vehicle vs. DNase I+cGAMP | 0 | 0.0050 |

Figure 6L

| | U | P value |
|---|---|---|
| DNase I+Vehicle vs. DNase I+cGAMP | 3 | 0.0152 |

Figure 6M (1d)

| | U | P value |
|---|---|---|
| DNase I+Vehicle vs. DNase I+cGAMP | 18.50 | 0.2233 |

Figure 6M (3d)

| | U | P value |
|---|---|---|
| DNase I+Vehicle vs. DNase I+cGAMP | 0 | 0.0007 |

Figure 6M (7d)

| | U | P value |
|---|---|---|
| DNase I+Vehicle vs. DNase I+cGAMP | 0 | 0.0002 |

Figure 7C (IL-6)

| | Z | P value |
|---|---|---|
| Sham vs. Vehicle | 3.731 | 0.0002 |
| Vehicle vs. DNase I | 2.109 | 0.0350 |

Figure 7C (TNF)

| | Z | P value |
|---|---|---|
| Sham vs. Vehicle | 3.461 | 0.0005 |
| Vehicle vs. DNase I | 2.379 | 0.0173 |

Figure 7E (IL-6)

| | U | P value |
|---|---|---|
| DNase I+Vehicle vs. DNase I+cGAMP | 0 | 0.0022 |

Figure 7E (TNF)

| | U | P value |
|---|---|---|
| DNase I+Vehicle vs. DNase I+cGAMP | 0 | 0.0020 |

Figure 8A

| | U | P value |
|---|---|---|
| Sham vs. SBI | 0 | 0.0022 |

Figure 8B

| | Z | P value |
|---|---|---|
| Sham vs. Vehicle | 3.871 | 0.0001 |
| Vehicle vs. Vitamin C 100 | 0.7381 | 0.4605 |
| Vehicle vs. Vitamin C 200 | 1.624 | 0.1044 |
| Vehicle vs. Vitamin C 500 | 3.608 | 0.0003 |

Figure 8C

| | Z | P value |
|---|---|---|
| Vehicle vs. Vitamin C 100 | 0.2042 | 0.8382 |
| Vehicle vs. Vitamin C 200 | 1.450 | 0.1472 |
| Vehicle vs. Vitamin C 500 | 3.165 | 0.0016 |

Figure 8D

| | Z | P value |
|---|---|---|
| Vehicle vs. Vitamin C 100 | 0.1021 | 0.9187 |
| Vehicle vs. Vitamin C 200 | 1.572 | 0.1159 |
| Vehicle vs. Vitamin C 500 | 2.899 | 0.0037 |

Figure 8F

| | Z | P value |
|---|---|---|
| Vehicle vs. Vitamin C 100 | 0.8165 | 0.4142 |
| Vehicle vs. Vitamin C 200 | 1.429 | 0.1530 |
| Vehicle vs. Vitamin C 500 | 3.633 | 0.0003 |

# Original Western Blot images

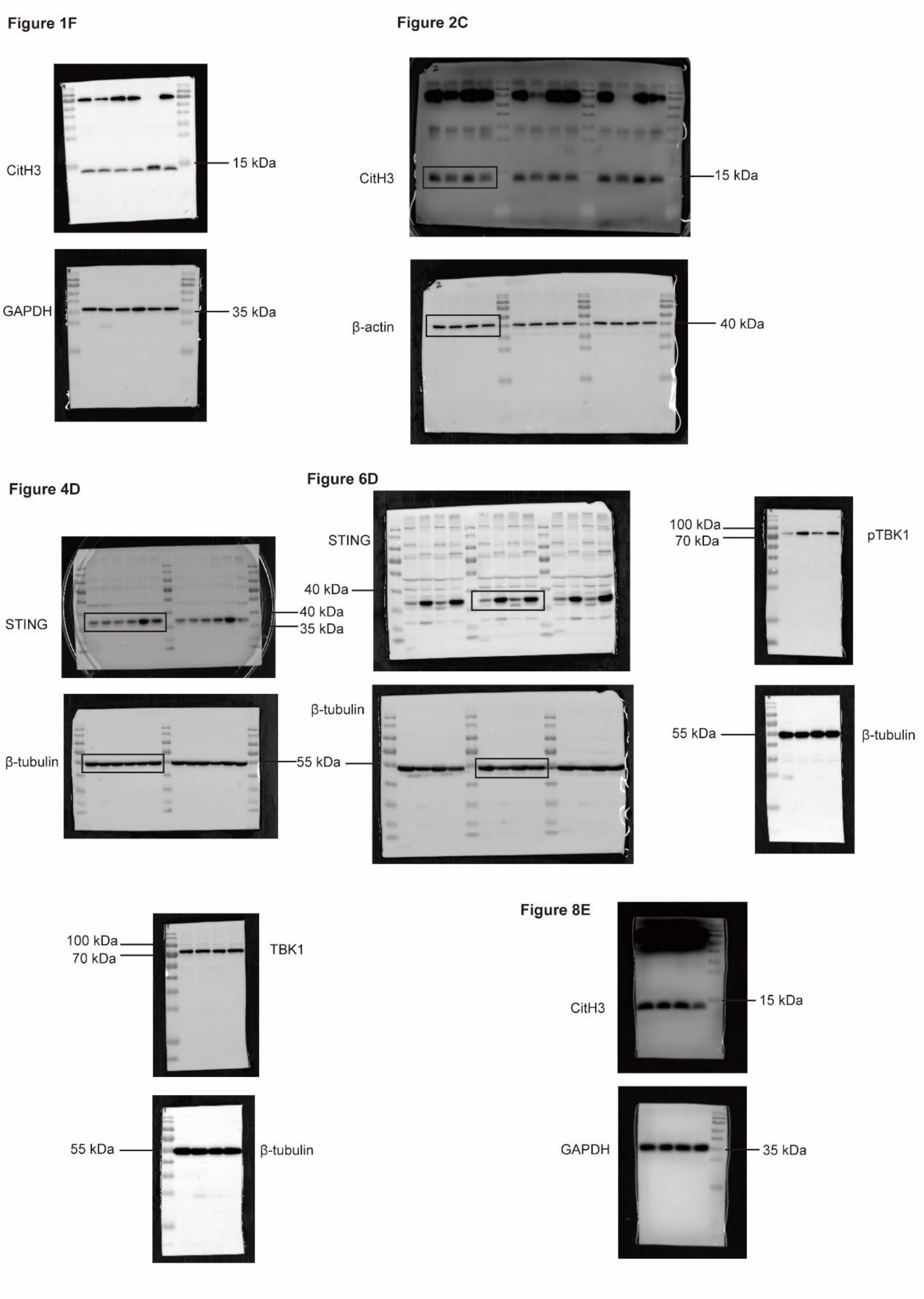

**References demonstrating the specificity of the primary antibodies**

**The primary antibodies:**

1) anti-CitH3(Kang et al. 2020)
2) anti-GAPDH (Wei et al. 2023)
3) anti-β-actin (#66009-1-Ig, Proteintech) (Lee et al. 2021)
4) anti-β-Tubulin (#66240-1-Ig, Proteintech) (Fish et al. 2021)
5) anti-STING (#19851-1-AP, Proteintech) (Pokatayev et al. 2020)
6) anti-pTBK1 (#AF8190, Affinity) (Zhao et al. 2022)
7) anti-TBK1 (#67211-1-Ig, Proteintech) (Chen et al. 2023)
8) anti-MPO (#sc-390109, Santa Cruz) (Hidalgo-Sastre et al. 2021)
9) anti-Iba-1(#17198, CST) (Banerjee et al. 2023)
10) anti-IL-1β (#sc-52012, Santa Cruz) (Wang et al. 2020)
11) anti-Iba-1 (#sc-32725, Santa Cruz) (Ardizzone et al. 2022)
12) anti-cGAS (#A8335, ABclonal) (Paul et al. 2022)
13) anti-GFAP (#3670, CST) (Nath and Julien 2023)

**References:**

Ardizzone A, Bova V, Casili G, Filippone A, Campolo M, Lanza M, Esposito E, Paterniti I (2022) SUN11602, a bFGF mimetic, modulated neuroinflammation, apoptosis and calcium-binding proteins in an in vivo model of MPTP-induced nigrostriatal degeneration. J Neuroinflammation 19 (1):107. doi:10.1186/s12974-022-02457-3

Banerjee S, Park T, Kim YS, Kim HY (2023) Exacerbating effects of single-dose acute ethanol exposure on neuroinflammation and amelioration by GPR110 (ADGRF1) activation. J Neuroinflammation 20 (1):187. doi:10.1186/s12974-023-02868-w

Chen NN, Zhang H, Zhu QS, Zeng T, Dai W, Zhou YL, Xin GF, Wu BD, Gong SJ, Jiang ZY, You QD, Xu XL (2023) Development of Orally Bioavailable Amidobenzimidazole Analogues Targeting Stimulator of Interferon Gene (STING) Receptor. J Med Chem 66 (8):5584-5610. doi:10.1021/acs.jmedchem.2c02046

Fish L, Khoroshkin M, Navickas A, Garcia K, Culbertson B, Hanisch B, Zhang S, Nguyen HCB, Soto LM, Dermit M, Mardakheh FK, Molina H, Alarcon C, Najafabadi HS, Goodarzi H (2021) A prometastatic splicing program regulated by SNRPA1 interactions with structured RNA elements. Science 372 (6543). doi:10.1126/science.abc7531

Hidalgo-Sastre A, Kuebelsbeck LA, Jochheim LS, Staufer LM, Altmayr F, Johannes W, Steiger K, Ronderos M, Hartmann D, Huser N, Schmid RM, Holzmann B, von Figura G (2021) Toll-like receptor 3 expression in myeloid cells is essential for

efficient regeneration after acute pancreatitis in mice. Eur J Immunol 51 (5):1182-1194. doi:10.1002/eji.202048771

Kang L, Yu H, Yang X, Zhu Y, Bai X, Wang R, Cao Y, Xu H, Luo H, Lu L, Shi MJ, Tian Y, Fan W, Zhao BQ (2020) Neutrophil extracellular traps released by neutrophils impair revascularization and vascular remodeling after stroke. Nat Commun 11 (1):2488. doi:10.1038/s41467-020-16191-y

Lee S, Karki R, Wang Y, Nguyen LN, Kalathur RC, Kanneganti TD (2021) AIM2 forms a complex with pyrin and ZBP1 to drive PANoptosis and host defence. Nature 597 (7876):415-419. doi:10.1038/s41586-021-03875-8

Nath B, Julien JP (2023) A New Mouse Model of Giant Axonal Neuropathy with Overt Phenotypes and Neurodegeneration Driven by Neurofilament Disorganization. J Neurosci 43 (22):4174-4189. doi:10.1523/JNEUROSCI.1959-22.2023

Paul S, Kaplan MH, Khanna D, McCourt PM, Saha AK, Tsou PS, Anand M, Radecki A, Mourad M, Sawalha AH, Markovitz DM, Contreras-Galindo R (2022) Centromere defects, chromosome instability, and cGAS-STING activation in systemic sclerosis. Nat Commun 13 (1):7074. doi:10.1038/s41467-022-34775-8

Pokatayev V, Yang K, Tu X, Dobbs N, Wu J, Kalb RG, Yan N (2020) Homeostatic regulation of STING protein at the resting state by stabilizer TOLLIP. Nat Immunol 21 (2):158-167. doi:10.1038/s41590-019-0569-9

Wang Y, Shi Y, Huang Y, Liu W, Cai G, Huang S, Zeng Y, Ren S, Zhan H, Wu W (2020) Resveratrol mediates mechanical allodynia through modulating inflammatory response via the TREM2-autophagy axis in SNI rat model. J Neuroinflammation 17 (1):311. doi:10.1186/s12974-020-01991-2

Wei W, Qin B, Wen W, Zhang B, Luo H, Wang Y, Xu H, Xie X, Liu S, Jiang X, Wang M, Tang Q, Zhang J, Yang R, Fan Z, Lyu H, Lin J, Li K, Lee MH (2023) FBXW7beta loss-of-function enhances FASN-mediated lipogenesis and promotes colorectal cancer growth. Signal Transduct Target Ther 8 (1):187. doi:10.1038/s41392-023-01405-8

Zhao C, Bao L, Qiu M, Wu K, Zhao Y, Feng L, Xiang K, Zhang N, Hu X, Fu Y (2022) Commensal cow Roseburia reduces gut-dysbiosis-induced mastitis through inhibiting bacterial translocation by producing butyrate in mice. Cell Rep 41 (8):111681. doi:10.1016/j.celrep.2022.111681

**Neurological function evaluation of each sub-test are described as follows.**

**(a) Spontaneous activity:** the animals were placed in their cage, and they were observed for 5 minutes. Scores indicate the following: (0) rat did not move at all; (1) rat barely moved; (2) rat approached 1–2 walls; (3) rat approached at least three walls of the cage.

**(b) Axial sensation:** Each side of the rat was stimulated using a cotton swab. The response of the rat was recorded. Scores indicate the following: (1) rat had a unilateral response; (2) asymmetric response; (3) rat had a brisk bilateral response.

**(c) Vibrissae touch:** A cotton swab was moved from the rear of the animal toward its head, touching the vibrissae gently. Scores indicate the following: (1) rat had a unilateral response; (2) asymmetric response; (3) rat equally turned head on both sides.

**(d) Limb symmetry:** The rat was suspended by the tail to assess movement of the limbs. Scores indicate the following: (0) no limb movement; (1) left limbs flexed; (2) asymmetric extension; (3) all limbs were extended symmetrically.

**(e) Lateral turning:** the rat was placed on a surface and allowed to roam. Scores indicate the following: (0) no turning at all; (1) unequal turning; (2) rat turned bilaterally less than 45◦ on both sides (3) rat turned bilaterally at least 45◦ on both sides.

**(f) Forefoot movement:** the rat was suspended by its tail allowing both forepaws to touch a flat surface. Scores indicate the following: (0) rat had a paretic forelimb; (1) rat walked in circles; (2) rat walked asymmetrically or to one side; (3) rat walked symmetrically on forepaws.

**(g) Climbing:** the rat was placed on a gripping surface at a 45◦ angle with the table. Scores indicate the following: (1) rat failed to climb or circled; (2) rat climbed but had a strong grip or rat climbed to the top and had a weak grip; (3) rat climbed to the top and had a strong grip.
